# Airway epithelial cell isolation techniques affect DNA methylation profiles with consequences for analysis of asthma related perturbations to DNA methylation

**DOI:** 10.1038/s41598-019-50873-y

**Published:** 2019-10-08

**Authors:** Rachel L. Clifford, Jamie Patel, Julia L. MacIsaac, Lisa M. McEwen, Simon R. Johnson, Dominick Shaw, Alan J. Knox, Tillie-Louise Hackett, Michael S. Kobor

**Affiliations:** 1 Nottingham NIHR Biomedical Research Centre, Nottingham MRC Molecular Pathology Node, Division of Respiratory Medicine, University of Nottingham, Nottingham University Hospitals NHS Trust, Nottingham, UK; 2 Centre for Molecular Medicine and Therapeutics, BC Children’s Hospital Research Institute, Department of Medical Genetics, University of British Columbia, Vancouver, British Columbia Canada; 3 Centre for Heart Lung Innovation, University of British Columbia, Vancouver, Canada; 4 Department of Anaesthesiology, Pharmacology & Therapeutics, University of British Columbia, Vancouver, Canada

**Keywords:** Asthma, DNA methylation

## Abstract

The airway epithelium forms the interface between the inhaled environment and the lung. The airway epithelium is dysfunctional in asthma and epigenetic mechanisms are considered a contributory factor. We hypothesised that the DNA methylation profiles of cultured primary airway epithelial cells (AECs) would differ between cells isolated from individuals with asthma (n = 17) versus those without asthma (n = 16). AECs were isolated from patients by two different isolation techniques; pronase digestion (9 non-asthmatic, 8 asthmatic) and bronchial brushings (7 non-asthmatic and 9 asthmatic). DNA methylation was assessed using an Illumina Infinium HumanMethylation450 BeadChip array. DNA methylation of AECs clustered by isolation technique and linear regression identified 111 CpG sites differentially methylated between isolation techniques in healthy individuals. As a consequence, the effect of asthmatic status on DNA methylation was assessed within AEC samples isolated using the same technique. In pronase isolated AECs, 15 DNA regions were differentially methylated between asthmatics and non-asthmatics. In bronchial brush isolated AECs, 849 differentially methylated DNA regions were identified with no overlap to pronase regions. In conclusion, regardless of cell isolation technique, differential DNA methylation was associated with asthmatic status in AECs, providing further evidence for aberrant DNA methylation as a signature of epithelial dysfunction in asthma.

## Introduction

The human airway epithelium represents the mucosal interface between the external environment and the lung. There is extensive evidence that the airway epithelium of individuals with asthma is abnormal including dysfunctional barrier function^1–4^, increased mucus secretion^5^, increased susceptibility to oxidative stress^6^, allergen^7^ and respiratory viruses^8^, along with altered baseline or stimulated cytokine and chemokine release^8–11^.

Epigenetics often refers to the study of modifications to DNA, and DNA packaging factors that do not alter the genetic sequence. Epigenetic regulatory mechanisms including histone modifications, non-coding RNA expression and DNA methylation, have been postulated to underlie epithelial dysfunction^12^. At a global level (not gene specific) the expression of epigenetic modifying enzymes and levels of histone 3 lysine 18 acetylation (H3K18Ac) and H3K9 methylation are different in airway epithelial cells isolated from non-asthmatic individuals compared to donors with asthma^13–15^. At a gene specific level, differential H3K18Ac is present at the promoter of the *ΔNp63*, *EGFR* and *STAT6* genes comparing airway epithelial cells from donors with and without asthma^15^, although the associated functional effect on gene expression was not assessed. More globally, miRNAs have been shown to be differentially expressed in airway epithelial cells from individuals with and without asthma^16,17^, and associate with differential gene expression, such as miR-449 negatively correlating with NOTCH1 expression^16^. Aberrant DNA methylation in cultured airway epithelial cells isolated from individuals with asthma has been identified in association with numerous genes including *KRT5*^18^, *STAT5A*^18^, *CRIP1*^18^ and *ADAM33*^19^. In freshly isolated airway epithelial cells, an extensive study of genome-wide DNA methylation and gene expression in over 100 individuals comparing individuals with and without asthma identified 40,892 CpGs differentially methylated between asthmatic and non-asthmatic donors, some of which were associated with changes in gene expression and linked to genetic variation (SNPs)^20^. Of 14 CpG sites associated with asthma in the whole blood of children, five were also differentially methylated in the nasal epithelium of the upper airway^21^. This evidence suggests that epigenetic processes are altered in the asthmatic epithelium. However, studies in cultured airway epithelial cells have performed limited quantification of DNA methylation, utilising small-scale microarray technologies and gene-targeted platforms. Studies of DNA methylation in freshly isolated cells, offer a valuable snapshot of the profile immediately following isolation, but do not allow for the characterisation of DNA methylation alterations that are maintained following removal from the native environment and are therefore potentially more persistent. Further, while there is a close relationship between the DNA methylation profiles of nasal and airway epithelia, significant differences are present^22^ and therefore while an easier cell source to obtain, changes to nasal epithelia may not be representative of changes occurring with lung disease.

In the current study we aimed to assess global DNA methylation in cultured human airway epithelial cells, isolated from 17 individuals with asthma and 16 individuals without asthma, to increase our understanding of potential perturbations of DNA methylation associated with asthma. Airway epithelial cells were isolated from patients by two different isolation techniques; pronase digestion (9 non-asthmatic, 8 asthmatic) and bronchial brush (7 non-asthmatic and 9 asthmatic). We identified significant effects of airway epithelial cell isolation technique on DNA methylation profiles, with consequences for downstream asthma analysis. However, we were able to identify significantly differentially methylated DNA regions between airway epithelial cells isolated from individuals with asthma versus those without, regardless of cell isolation technique, highlighting the intricate relationship between asthma status and DNA methylation.

## Results

### Cohort characteristics

Airway epithelial cells were isolated from 17 asthmatic individuals and 16 non-asthmatic individuals. Cells were isolated by either pronase digestion or bronchial brushings. Airway epithelial cells were isolated by pronase digestion from nine non-asthmatic and eight asthmatic individuals and by bronchial brush from seven non-asthmatic individuals and nine asthmatic individuals. On no occasion were both isolation techniques used for the same individual. For initial analysis of the effect of isolation technique on DNA methylation profiles, only samples from non-asthmatic individuals were used. Nine pronase isolated samples and seven bronchial brush isolated samples were included in the analysis. Samples were matched for sex, age and smoking status (Table 1). For analysis of the effect of asthmatic status on DNA methylation, samples were separated by isolation technique. Cells were isolated by pronase digestion from nine individuals without asthma and eight individuals with asthma. Samples were matched for sex, age and smoking status (Table 2). Cells were isolated by bronchial brush from seven individuals without asthma and nine individuals with asthma. Samples were matched for sex and smoking status (Table 3), however age was significantly different between individuals with and without asthma and included as a covariate in models accordingly.

**Table 1:** Pronase digestion versus bronchial brush isolation healthy donor demographics.

N	Pronase Digested	Bronchial Brush	p-value
	9	7	
Age (mean ± SEM)	21 ± 3.27	31.85 ± 5.00	0.0798 (t-test)
Female/Male	2/7	2/5	>0.999 (Fishers exact)
Smoker (current/non)	2/7	0/7	0.4750 (Fishers exact)

**Table 2:** Pronase digestion isolation from individuals with and without asthma donor demographics.

N	Non-asthmatic	Asthmatic	p-value
	9	8	
Age (mean ± SEM)	21 ± 3.27	18.63 ± 3.173	0.6118 (t-test)
Female/Male	2/7	6/2	0.0567 (Fishers exact)
Smoker (current/non)	2/7	2/6	>0.9999 (Fishers exact)

**Table 3:** Bronchial brush isolation from individuals with and without asthma donor demographics.

N	Non-asthmatic	Asthmatic	p-value
	7	9	
Age (mean ± SEM)	56.22 ± 3.8	31.85 ± 5.0	0.0015 (t-test)
Female/Male	2/5	5/4	0.3575 (Fishers exact)
Smoker (current/non)	0/7	3/6	0.2125 (Fishers exact)

### Airway epithelial cell DNA methylation varied by cell isolation technique

**Figure 1:**
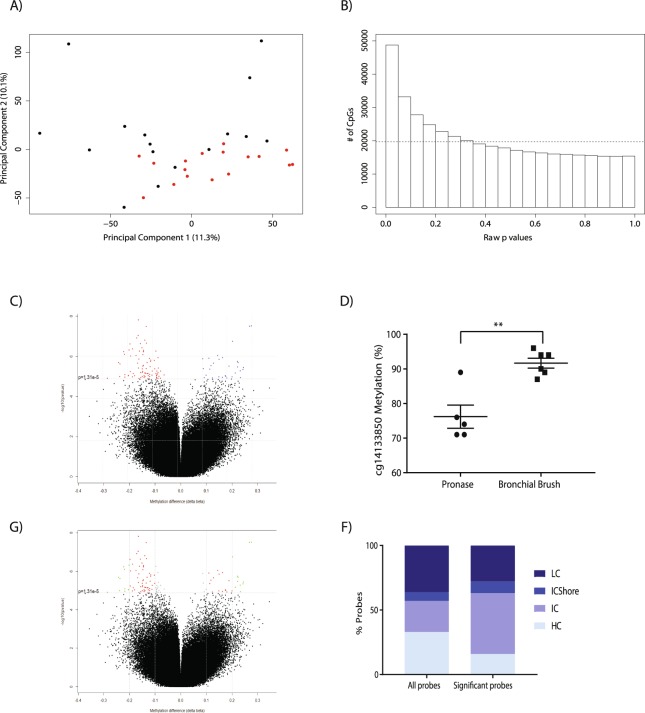
Differential DNA methylation between airway epithelial cells isolated via pronase versus bronchial brush. (**A**) Multidimensional scale plot of samples used in the study. Black point represent bronchial brush isolated cells, red points represent pronase isolated cells. (**B**) P value distribution for the effect of isolation technique. Dashed line indicates the distribution expected by chance. (**C**) Plot of the 413,350 CpG probes used in the analysis. Red/Blue points = Benjamini-Hochberg adjusted p value < 0.05. Red = negative delta beta, Blue = positive delta beta. (**D**) Pyrosequencing of cg14133850; **p = 0.0014 unpaired t-test. (**E**) Plot of the 413,350 CpG probes used in the analysis. Grey/red/green points = Benjamini-Hochberg p value < 0.05. Grey = delta beta < 0.1, red = delta beta > 0.1, green = delta beta > 0.2. (**F**) Gene feature type for all analysis probes and all significant probes. HC = high-density CpG islands (CG content > 55%, Obs/Exp CpG ratio > 0.75 and length > 500 bps), IC = intermediate-density CpG islands (CG content > 50%, Obs/Exp CpG ratio > 0.48 and length > 200 bps), LC = low-density CpG regions (non-islands), ICShore = regions of intermediate-density CpG island that border HCs.

Principal component analysis was used to explore global patterns of variation in DNA methylation at over 485,000 CpG sites. A prominent difference in clustering between how the airway epithelial cells were isolated, pronase digestion or physical brushing and washing, was observed (Fig. 1A). To understand whether we could consider cells isolated by bronchial brush or pronase digestion within the same analysis for alterations associated with asthmatic status, we compared single site resolution DNA methylation in airway epithelial cells isolated from healthy individuals via bronchial brush versus pronase digestion, by linear modelling. The demographics of the individuals from which the cells were isolated are shown in Table 1. The deviation of the nominal P value distributions from random suggested an association of isolation method and DNA methylation in airway epithelial cells (Fig. 1B). Specifically, we identified 111 CpGs with significantly different CpG methylation between collection techniques (Benjamini-Hochberg p < 0.05) (Fig. 1C, blue and red points). To confirm the array data differential methylation of cg14133850 was verified by pyrosequencing (Fig. 1D). The 111 CpGs were associated (as defined by Illumina annotation to the closest transcription start site) with 103 different genes (Supplementary Table S1) and four genes were associated with more than one CpG probe (*CHRNE*, *EDAR*, *GALNT9*, *LOC149837*; Bold highlight in Supplementary Table S1). 81 CpG sites were more methylated in bronchial brush isolated cells compared to pronase isolated cells (Fig. 1C, red points), while 30 CpG sites were more methylated in pronase isolated cells than bronchial brush isolated cells (Fig. 1C, blue points). In terms of effect size, 91 CpG sites displayed a beta difference (delta beta, Δβ) of greater than 0.1 which approximates to a 10% difference in CpG methylation (Fig. 1E, red points). 22 CpG sites displayed a Δβ of greater than 0.2 (20% difference) in CpG methylation (Fig. 1E, green points) with a maximum Δβ of 0.28. 20 CpG sites displayed a methylation difference less than 10% (Fig. 1E, grey points) with a minimum Δβ of 0.06. The 111 differentially methylated CpG probes were enriched for DNA regions of intermediate CpG density, which differed from the distribution of the full analysis probes set (χ^2^ P value = 0.0022) (Fig. 1F).

**Figure 2:**
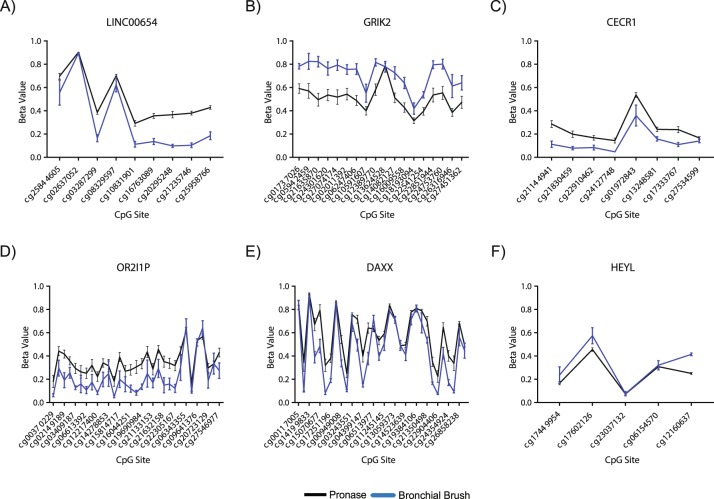
Regional differential DNA methylation between airway epithelial cells isolated via pronase versus bronchial brush. DNA methylation in pronase and bronchial brush isolated airway epithelial cells at differentially methylated regions associated with (**A**) *LINC00654*, (**B**) *GRIK2*, (**C**) *CECR1*, (**D**) *OR2I1P*, (**E**) *DAXX* and (**F**) *HEYL*.

Subsequently, we were interested in whether a regional analysis of differential DNA methylation would highlight further differential DNA methylation associated with airway epithelial isolation technique. DMRcate regional analysis^23^ identified nine differentially methylated regions (Table 4), seven of which contained three or more CpG sites. Of these seven, six were annotated to the following genes: *LINC00654* (Fig. 2A), *GRIK2* (Fig. 2B), *CECR1* (Fig. 2C), *OR2I1P* (Fig. 2D), *DAXX* (Fig. 2E), and *HEYL* (Fig. 2F). Only a single CpG, cg09495303 associated with the gene *CHRNE*, was identified by both linear modelling and DMRcate regional analysis, suggesting the 111 linear modelling CpGs were dispersed throughout the genome and that regional analysis had the capacity to identify further differences in DNA methylation by maximising statistical power.

**Table 4:** Summary of the Nine DNA regions differentially methylated between pronase and bronchial brush isolated airway epithelial cells.

Chromosome Location	Start Location	End Location	Region Width	Number of CpGs	Minimum FDR	Max. difference in β value	Mean difference in β value	Overlapping Promoters
chr20	5485144	5485873	730	9	2.37E-76	0.28	0.18	LINC00654
chr17	4804674	4804838	165	2	2.97E-21	−0.17	−0.16	CHRNE, C17orf107
chr6	101846409	101847706	1298	18	9.24E-33	−0.33	−0.21	GRIK2
chr22	17680477	17680706	230	8	7.21E-19	0.18	0.11	CECR1
chr2	15804917	15805012	96	3	2.28E-19	−0.13	−0.08	NA
chr6	134210279	134210307	29	2	1.15E-18	0.09	0.08	TCF21
chr6	29520965	29521803	839	31	1.77E-30	0.23	0.13	OR2I1P
chr6	33287809	33289280	1472	32	2.85E-55	0.31	0.13	DAXX
chr1	40105664	40105764	101	5	1.93E-19	−0.16	−0.07	HEYL

These data suggested that the DNA methylation profiles of airway epithelial cells isolated via pronase digestion and bronchial brush were different and therefore needed to be considered as distinct sample types for disease association analyses.

### Differential DNA methylation is associated with asthma status

To identify differences in DNA methylation in association with asthmatic status, we performed separate DNA methylation analyses on DNA from airway epithelial cells isolated by pronase digestion or bronchial brushings.

**Figure 3:**
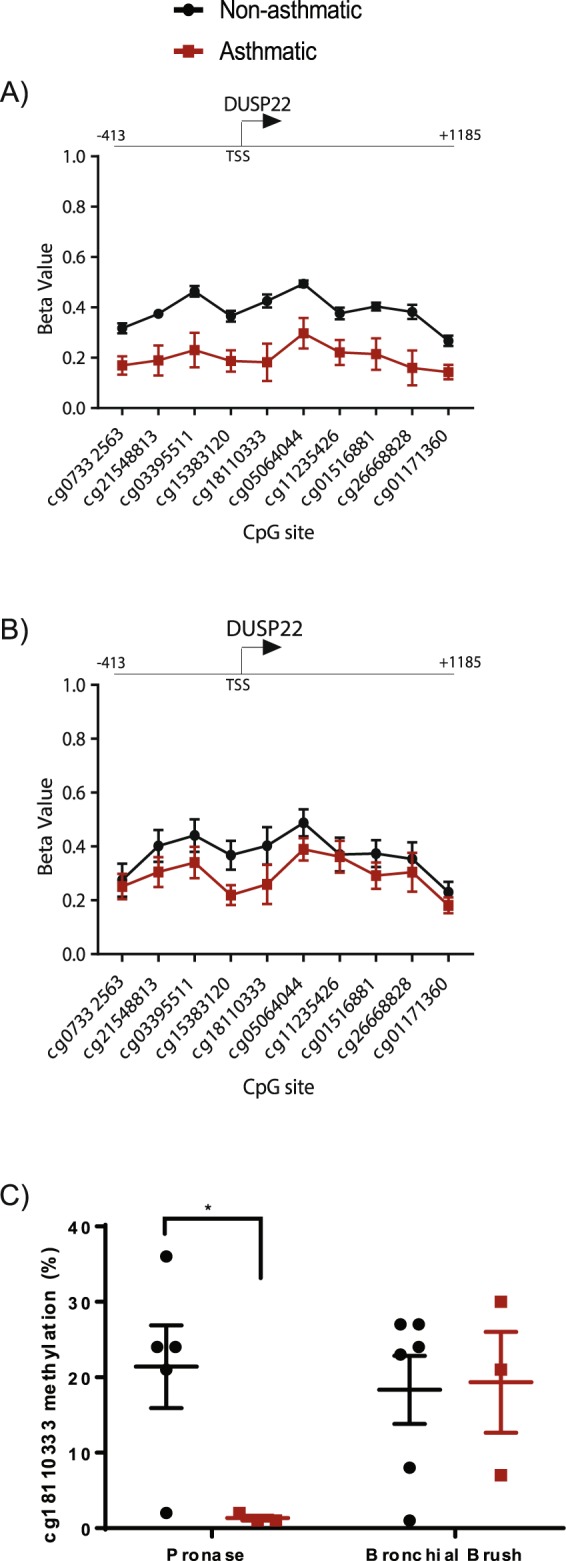
DNA region displaying a maximum difference in DNA methylation of 20% between airway epithelial cells isolated via pronase digestion from individuals with and without asthma. (**A**) Methylation level of 10 CpGs associated with *DUSP22* in pronase isolated HBEC cells from individuals with and without asthma. (**B**) Methylation level of 10 CpGs associated with *DUSP22* in bronchial brush isolated HBEC cells from individuals with and without asthma. (**C**) Pyrosequencing validation of differential DNA methylation at cg18110333 between cells isolated from asthmatic and non-asthmatic individuals by pronase digestion but not bronchial brush isolation. ^*^p value = 0.03 (t-test).

First, we compared single site DNA methylation by linear modelling in pronase digestion isolated cells. The demographics of the individuals from which the pronase-derived cells were isolated are shown in Table 2. No individual differentially methylated CpG sites were identified (Benjamini-Hochberg p < 0.05), so we assessed differential methylation on aggregated sites using the regional DNA methylation R package *DMRcate*, including all CpGs specified by a nominal p value limit of <0.005. 15 differentially methylated regions were identified in cells isolated from individuals with asthma as compared to cells from individuals without asthma (Table 5), 14 of which contained at least three CpG sites. Ten regions were annotated to a known gene and a single region, associated with the promoter region of *DUSP22*, and displayed a maximum difference in DNA methylation (i.e. at least one probe displayed a mean difference in methylation) of 20% (Δβ = 0.2) (Fig. 3A). The maximum Δβ of 0.243 was at *DUSP22* cg18110333 and signifies an average decrease in methylation of 24.3% at cg18110333 in pronase isolated airway epithelial cells isolated from individuals with asthma versus those without asthma. The mean difference in DNA methylation between pronase isolated airway epithelial cells isolated from individuals with asthma versus those without asthma, across the *DUSP22* region of 1599 bp and including ten array-CpGs was 18.7%. Differential DNA methylation of the same region was not identified in airway epithelial cells isolated from individuals with and without asthma via bronchial brushing (Fig. 3B). Differential methylation of cg18110333 in pronase but not bronchial brush isolated airway epithelial cells was verified by pyrosequencing (Fig. 3C).

**Table 5:** Summary of the 15 DNA regions differentially methylated between pronase isolated airway epithelial cells isolated from donors with and without asthma.

Chromosome Location	Start Location	End Location	Region Width	Number of CpGs	Minimum FDR	Max. difference in β value	Mean difference in β value	Overlapping Promoters
chr20	32856747	32856898	152	2	0.0024633	0.08	0.08	NA
chr10	44197967	44198369	403	3	0.0002902	−0.14	−0.12	NA
chr10	133995286	133995387	102	3	0.0024633	0.11	0.08	NA
chr2	133402827	133403009	183	3	0.0002206	−0.09	−0.08	NA
chr3	195578240	195578280	41	3	0.0047179	0.15	0.12	NA
chr6	152125861	152126092	232	3	0.0015607	0.15	0.10	ESR1
chr6	31238388	31239767	1380	15	2.818E-09	−0.16	−0.04	HLA-C
chr2	3642098	3642967	870	11	4.476E-05	0.11	0.06	COLEC11
chr7	117067136	117067978	843	11	8.756E-05	−0.12	−0.05	ASZ1
chr6	291687	293285	1599	10	1.869E-21	0.24	0.19	DUSP22
chr15	91473059	91473569	511	8	0.0006853	−0.08	−0.04	UNC45A
chr3	42977777	42978180	404	7	0.0001463	−0.10	−0.07	KRBOX 1
chr14	37135384	37136283	900	6	3.509E-05	0.10	0.08	PAX9
chr2	66659348	66659590	243	4	0.0010565	−0.17	−0.10	MEIS1
chr7	128696069	128696229	161	4	0.0006638	−0.10	−0.05	TNPO3

**Figure 4:**
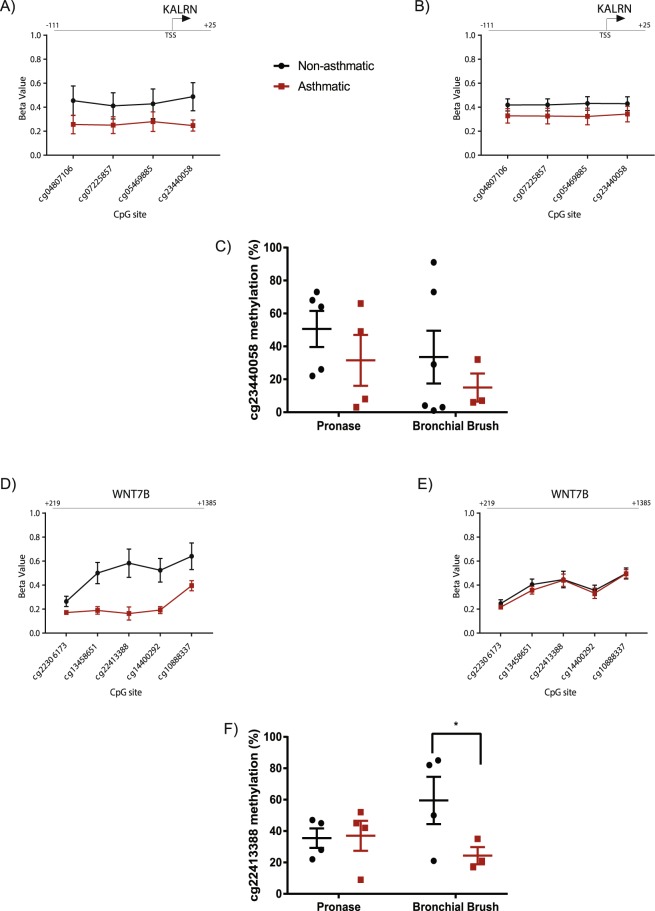
DNA region displaying a maximum difference in DNA methylation of 40% between airway epithelial cells isolated via bronchial brush from individuals with and without asthma. (**A**) Methylation level of four CpGs associated with *KALRN*, identified by *DMRcate*, in bronchial brush isolated airway epithelial cells from individuals with and without asthma. (**B**) Methylation level of four CpGs associated with *KALRN* in pronase isolated HBEC cells from individuals with and without asthma. (**C**) Pyrosequencing of cg2344005 in cells isolated from asthmatic and non-asthmatic individuals by bronchial brush and pronase digestion. (**D**) Methylation level of five CpGs associated with *WNT7B*, identified by *DMRcate*, in bronchial brush isolated airway epithelial cells from individuals with and without asthma. E) Methylation level of five CpGs associated with *WNT7B* in pronase isolated HBEC cells from individuals with and without asthma. (**F**) Pyrosequencing verification of differential DNA methylation at cg22413388 between cells isolated from asthmatic and non-asthmatic individuals by pronase digestion but not bronchial brush isolation; ^*^t-test p value = 0.04.

Subsequently, we compared single site DNA methylation by linear modelling in airway epithelial cells isolated by bronchial brushings. The demographics of the individuals from which the bronchial brush derived cells were isolated are shown in Table 3. As age was significantly different between our samples isolated from individuals with and without asthma we adjusted for age as a covariate. Similar to the pronase digested analysis, no individually differentially methylated CpG sites were identified (Benjamini-Hochberg p < 0.05) so we also assessed differential methylation on aggregated sites using *DMRcate*, including all CpGs specified by a nominal p value limit of <0.005 and adjusting for age as a covariate. 849 differentially methylated regions were identified between the asthma and non-asthma groups (Supplementary Table S2), 615 of which contained at least three CpG sites and 427 regions were annotated to a known gene. In terms of effect size, 212 of these regions displayed a maximum difference in DNA methylation of greater than 20%, with 68 displaying an average difference in DNA methylation of > 20% between airway epithelial cells isolated from individuals with asthma versus those without asthma. In particular, two regions associated with the *KALRN* (Fig. 4A–C) and *WNT7B* (Fig. 4D–F) promoters had a difference in DNA methylation between airway epithelial cells isolated from individuals with asthma versus those without asthma of greater than 40% (mean Δβ 0.46 and 0.45 respectively). For the *KALRN* associated region the maximum Δβ was at cg23440058 and signified a decrease in methylation of 48% at cg23440058 in airway epithelial cells isolated from individuals with asthma versus those without asthma. However, differential methylation of this CpG did not validate by pyrosequencing (Fig. 4C). For the *WNT7B* associated region the maximum Δβ of 0.66 was at cg22413388 and signified a decrease in methylation of 66% at cg22413388 in airway epithelial cells isolated from individuals with asthma versus those without asthma, as validated by pyrosequencing (Fig. 4F). The mean difference in methylation between airway epithelial cells isolated from individuals with asthma versus those without asthma across the WNT7B region of 1167 bp and including five array CpGs was 45%.

Differential DNA methylation of the same *KALRN* and *WNT7B* associated regions were not identified in airway epithelial cells isolated from individuals with and without asthma via pronase digestion (Fig. 4B,E respectively). Indeed, there were no regions that were differentially methylated in association with asthma status in both pronase and bronchial brush isolated airway epithelial cells. Furthermore, analysis of association of DNA methylation perturbations with asthmatic status in the full dataset, including both pronase and bronchial brush isolated cells, identified 28 differentially methylated regions (Supplementary Table S3) of which only two were in common with one of the 15 pronase isolated epithelial cells regions (Italic and underlined text in Supplementary Table S3) and only eight were in common with one of the 849 bronchial brush isolated epithelial cells regions (Bold text in Supplementary Table S3), suggesting asthma associated regions would not have been identified in a combined analysis where no attention to isolation technique has been taken. In the majority of cases the combined analysis identified regions of equal or smaller size than the separate analyses, with smaller maximum and mean differences in DNA methylation (Supplementary Table S4). For the region associated with SP6, the direction of change of DNA methylation was different; a reduction in DNA methylation in cells isolated from individuals with asthma in bronchial brush isolated cells versus an increase in DNA methylation in the combined dataset (Supplementary Table S4). Finally, all nine differentially methylated regions identified between the two isolation techniques were identified as associated with asthmatic status in the combined analysis (Blue font in Supplementary Table S3), representing false positive asthma associated regions based on underlying cell isolation artefact. This highlights the importance of identifying the two separate cell populations and handling them appropriately in separate analyses.

## Discussion

The main finding of this study is that asthmatic status is associated with differential DNA methylation in airway epithelial cells, a finding that further confirms previous observations^18–21^. Furthermore, in this study we identified differential DNA methylation between cells isolated via bronchial brush and pronase digestion. Subsequently, we showed that different DNA regions are differentially methylated in association with asthmatic status in the cells isolated via pronase versus bronchial brush isolation. This highlights the complexity of the asthmatic epithelium and indicates the care required when working with cell types in complex airway diseases.

The difference in DNA methylation due to isolation technique is an important observation that needs to be considered in future studies of not only epigenetic processes but also studies of airway epithelial cell function. The airway epithelium in the larger conducting airways, accessible to bronchiole brushings is pseudostratified and contains a mixed population of individual cell types. DNA methylation profiles are known to be highly cell type specific. While there have been no studies to assess the DNA methylation profiles of individual cell types within the airway epithelium, and the current study does not allow us to say with certainty, it is plausible that the differences we observe in DNA methylation between the pronase and the bronchial brush isolations represents different populations of cells being sampled. For instance, pronase digestion collects all cells down to the basement membrane including basal cells, while a bronchial brush is likely to only collect cells at the apical surface. Here, we identified 111 CpGs that significantly differed between pronase digestion isolated airway epithelial cells and bronchial brush isolated cells. Genes associated with these CpGs include genes linked to cell- and tight- junction formation including *MPP7* (Membrane Palmitoylated Protein 7), *TJAP1* (Tight Junction Associated Protein 1) and *CLDN15* (Claudin 15), potentially linking the two cell populations to different epithelial membrane functions. Further studies utilising single cell bisulfite sequencing to fully profile the airway epithelium would be of value.

The analysis to identify differential DNA methylation in association with asthmatic status did not identify any individually significant CpG sites, likely due to the diminished statistical power resulting from splitting the data set by isolation technique. However, we did identify differentially methylated regions in association with asthma status in both pronase and bronchial brush isolated airway epithelial cells. Despite a similar sample size, a much larger number of differentially methylated DNA regions were identified in bronchial brush isolated cells (15 regions in pronase compared to 849 in bronchial brush). The definitive reason for this is unknown. It is unlikely to be due to different severities of asthma in the bronchial brush versus pronase isolated cells as both data sets included a range of asthma severity from mild to severe. A possible explanation may be that a higher proportion of apical cells in the bronchial brush population have greater exposure to environmental insults over time which may result in greater aberration than the basal cells of the pronase digested cells. Similarly, the overall higher age of the donors from which the epithelial cells were isolated by bronchial brushings may have given more time for environmental-associated methylation modifications to occur to the cellular DNA. In support of our finding that pronase and bronchial brush isolated cells exhibit different DNA methylation profiles in association with asthma, of the 4187 CpGs in the 849 bronchial brush regions, 101 CpGs were also identified by Nicodemus-Johnson *et al*.^20^, in freshly isolated bronchial brush airway epithelial cells, while only a single CpG, cg09646983, from the pronase isolated airway epithelial cell regions was identified in both analyses. Neither the pronase or bronchial brush isolation technique identified the 5 CpGs associated with asthma in nasal epithelial cells^21^, suggesting asthma-associated perturbations in DNA methylation may differ between upper (nasal) and lower airway epithelial cells.

Pyrosequencing validation of the asthmatic bronchial brush region associated with *KALRN* did not show a significant asthmatic versus non-asthmatic difference in methylation at the individual CpG. Considering the regional plot of this gene (Fig. 4A), it is not surprising that it was only upon combined power of a regional analysis that the region was identified. In support of this, a linear modelling p value distribution of the bronchial brush isolated asthma analysis (not shown) displayed a right hand skew, indicative of CpGs with differences in methylation in association with asthmatic status but below multiple test significance due to power.

The genes associated with the largest difference in DNA methylation between cells isolated from individuals with asthma versus those without asthma (pronase, *DUSP22*; bronchial brush, *WNT7B*) have links to asthma pathogenesis. *DUSP22* (dual specificity phosphatase 22) is differentially methylated between umbilical cord white blood cell samples from children with high versus low maternal polycyclic aromatic hydrocarbon exposure suggesting its DNA methylation may be modulated by exposure to traffic-related air pollution^24^, a major risk factor for asthma development and exacerbation. Whereas, Wnt signalling is important in lung airway differentiation and Wnt7b is expressed in the airway epithelium where it plays a critical role in lung airway development^25,26^ and airway repair in the adult^27^, dysfunction of which potentially contributes to asthma pathogenesis.

A limitation to the current study is the inability to assess whether any of the differences in DNA methylation observed are associated with or due to medication regimens of the individuals involved. We had limited data available for medication taken by the individuals in the current study preventing us from addressing the question and the effect of common asthma medications on airway epithelial cell DNA methylation represents a valid area for future study. We also did not have cells isolated using both techniques from the same individual to confirm our findings. Once isolated the cells were grown in the same tissue culture media and under the same conditions until they were processed for DNA isolation however, subsequent studies to more fully characterise DNA methylation profiles of different airway epithelia offer an important opportunity. Finally, for technical reasons DNA was isolated from cells at passage 4, the highest passage recommended for airway epithelial cells. Although a full analysis of passage effects was not possible a limited number of within donor, passage replicates were included in the dataset and showed high correlation, reducing concern of global disruption to DNA methylation due to passage effects.

In conclusion, by assessing global DNA methylation in airway epithelial cells we have identified a number of gene regions displaying differential DNA methylation associated with asthma. Although the differentially methylated regions identified were unique, the association with asthma was maintained regardless of the isolation technique used to obtain airway epithelial cells. This raises the importance of attention to detail when isolating cells known to be a heterogeneous population, for *in vitro* studies, and suggests pronase isolated epithelial cells may have limited utility for DNA methylation analysis. However emerging single cell technologies will offer new opportunities around heterogenous sample types. Finally, our study highlights the consistency of the finding that aberrant DNA methylation in airway epithelial cells is associated with asthmatic status and is therefore a signature underlying the pathogenesis of asthma.

## Methods

### Cell isolation and culture

Airway epithelial cells were isolated by pronase digestion from proximal airways of non-transplantable human donor lungs of subjects with and without asthma and was approved by the Providence Health Care Research Ethics Board (H13-02173), University of British Columbia. Airway epithelial cells were also isolated from proximal airway bronchial brushings from individuals with and without asthma and was approved by the East Midlands Research Ethics Committee (08/H0407/1), University of Nottingham. Informed consent was obtained from all donors and experiments were performed in accordance with relevant institutional guidelines and regulations. Briefly, for pronase isolation as previously described^28,29^, 2–4 cm airway segments were washed in PBS and placed in Bronchial Epithelial Basal Media (BEBM; Lonza, Basel, Switzerland) containing 1.4 mg/ml pronase for 16 h at 4 °C. Airway sections were then washed in BEBM to aid cell dissociation and the harvested cell suspension was passed through a 70 μm nylon mesh. Pronase was neutralized after which the cell suspension was collected and resuspended in Bronchial Epithelial Growth Medium (BEGM; Lonza, Basel, Switzerland) including SingleQuots containing Bovine Pituitary Extract [BPE], Hydrocortisone, human Epidermal Growth Factor (hEGF), Epinephrine, Transferrin, Insulin, Retinoic Acid, Triiodothyronine, and Gentamicin/Amphotericin-B. Bronchial brushes were agitated in BEGM and disrupted cells were transferred to 6 well plates for adherence. For this study all cells, both pronase and bronchial brush isolated cells, were grown in 6-well plates in culture at 37 °C in 95% air and 5% CO_2_ in BEGM, under identical conditions and used at passage 4 for all experiments. Cells were serum deprived in BEBM for 24 hours prior to DNA and RNA extraction. Donor demographics are listed in Tables 1, 2 and 4.

### DNA isolation

DNA was isolated from samples collected using the AllPrep DNA/RNA Mini Kit (Qiagen) as per the manufacturer’s instructions.

### Bisulfite conversion and DNA methylation arrays

750 ng of purified genomic DNA was bisulfite converted using the EZ DNA Methylation Kit (Zymo Research) as per the manufacturer’s instructions. Specific incubation conditions for the Illumina Infinium Methylation Assay were used as per the manufacturer’s protocol Appendix. Samples were eluted in 12 µl. Bisulfite-converted DNA was assessed for concentration and quality using a NanoDrop^TM^ 8000 Spectrophotometer (Thermo Fisher Scientific), and 160 ng of the conversion product was used for genome-wide DNA methylation evaluation at over 485,000 CpG sites using the Illumina Infinium HumanMethylation450 BeadChip array, according to the manufacturer’s protocol.

### Data quality control and normalisation

Raw intensity data files obtained from the HiScan (Illumina) were imported into the R statistical software (version 3.2.1) using the minfi package (v. 1.14.0)^30^. Probes with detection P values greater than 0.01, probes with missing beta values, and probes with fewer than 3 beads contributed to the signal in 1 or more samples were excluded (32,350 probes removed). A total of 11,648 probes residing on the X chromosome or the Y chromosome were also removed to control for sex-derived differences on the array. Finally, probes known to be polymorphic at the CpG locus or to cross-hybridize, and those examining single nucleotide polymorphisms were removed (28,164 probes removed)^31^. The final number of probes remaining was 413,350 for downstream analyses. The final probe set was normalized using the funtooNorm algorithm^32^, which extends the funNorm procedure^33^ and is purported to correct for unwanted variation whilst preserving important differences in DNA methylation patterns between different cell types. We employed the normalization option of principal components regression with 5 principal components. Two values of DNA methylation were calculated, beta-values (β-values) and M-values. β-values are the ratio of all methylated probe intensities over total signal intensities (methylated and unmethylated) and have a range from 0 to 1, approximately representing percent methylation. M-values are the log transformation of β-values and are more statistically robust^34^. All statistical analyses were performed using M-values and β-values were used for visualisation and discussion purposes. Within donor, passage replicates showed high correlation; Donor A p2 vs p3 – r2 = 0.9876, Donor B p2 vs p4 – r2 = 0.9821, Donor C p0 vs p1 – r2 = 0.9902), reducing concern of global disruption to DNA methylation due to passage effects.

### Differential DNA methylation analysis

All statistical analyses were performed using R statistical software. Probes with DNA methylation levels significantly different between pronase isolated and bronchial brush isolated airway epithelial cells in non-asthmatic individuals were identified using the *limma* package in R^35^ followed by control for false discovery rate via the Benjamini–Hochberg procedure^36^. Within isolation technique, individual probes with DNA methylation levels significantly different between airway epithelial cells isolated from donors with and without asthma were also identified using the *limma* package. For bronchial brush analyses, sample age was included as a covariate. As no independently significant sites were identified as associated with asthma status using *limma*, we looked at aggregated sites to identify differentially methylated regions (DMRs) using the *DMRcate* package in R^23^, which uses Guassian kernel smoothing to find patterns of differential methylation, agnostic to genomic annotation. We used the authors’ recommended bandwidth (λ) of 1,000 base pairs, scaling factor (C) of 2 and nominal p-value cutoff of P < 0.005.

### Bisulfite PCR-pyrosequencing

Bisulfite PCR-pyrosequencing was used to validate differences in DNA methylation at *MPP7* CpG cg14133850, DUSP22 CpG cg18110333, *KALRN* CpG cg23440058 and *WNT7B* CpG cg22413388. Bisulfite PCR-pyrosequencing assays were designed with PyroMark Assay Design 2.0 (Qiagen). The regions of interest were amplified by PCR using the HotstarTaq DNA polymerase kit (Qiagen) as follows: 15 minutes at 95 °C (to activate the Taq polymerase), 45 cycles of 95 °C for 30 s, 55 °C for 30 s, and 72 °C for 30 s, and a 5 minute 72 °C extension step. For pyrosequencing, single-stranded DNA was prepared from the PCR product with the Pyromark™ Vacuum Prep Workstation (Qiagen) and sequencing was performed using sequencing primers on a Pyromark™ Q24 pyrosequencer (Qiagen). The quantitative levels of methylation for each CpG dinucleotide were calculated with Pyromark Q24 software (Qiagen). Primer sequences were: cg22413388 - Forward_Bio AGGGAGAGGGTGTGTGAGT, Reverse CCAACCAAAAACCCCTCCCCATATTA, Sequencing ATACCATACCACACTTAAA; cg23440058 -Reverse_Bio TCTAAACCAACTACTCTACTACTTCTAA, Forward TTGTTTTATTGTTGTTGTTGGTAGAA, Sequencing TGTTGTTGTTGGTAGAAT; cg18110333 - Reverse_Bio AACCCAAAACCCTCCAAAAACCC, Forward GGTATAGGTTTAGGTGTTTTTGTT, Sequencing GGTTTTAGTAGGGAAGTTTTAG; cg14133850 - Reverse_Bio AACTAACCAATCCAATCATTTTTCCCATAA, Forward GGTGTTTTGTTTTTGTGGTATAAGG, Sequencing GTTTTTTTGGAATTAATGGG.

## Supplementary information

Supplementary Information

## Data Availability

The datasets analysed during the current study are available in the Gene Expression Omnibus repository, https://www.ncbi.nlm.nih.gov/geo/query/acc.cgi?acc=GSE137716 GEO-REF:GSE137716.
