# Supplementary material for: Airway epithelial cell isolation techniques affect DNA methylation profiles with consequences for analysis of asthma related perturbations to DNA methylation
Source: Sci Rep. 2019 Oct 8;9:14409. doi: 10.1038/s41598-019-50873-y (PMC6783553; doi:10.1038/s41598-019-50873-y)
Supplement: Supplementary file 1 — Supplementary Information [file 41598_2019_50873_MOESM1_ESM.pdf]

Supplementary Information

Airway epithelial cell Isolation technique affects DNA methylation profiles with consequences for analysis of asthma related perturbations to DNA methylation.

Rachel L. Clifford[1,2*], Jamie Patel[1], Julia L. MacIsaac[2], Lisa M. McEwen[2], Simon Johnson[1], Dominick Shaw[1], , Alan J. Knox[1], Tillie-Louise Hackett[3,4, #], Michael S. Kobor[2,#].

S1 Table: 111 CpG sites differentially methylated between airway epithelial cells isolated via pronase digestion versus bronchial brushing.

Probe ID	P Value	Benjamini Hochberg Adjusted P Value	Difference in beta value (pronase reference)	CpG Class	Distance to closest TSS	Closest TSS gene name
cg12160637	1.54E-08	0.00331934	-0.16	HC	-358	HEYL
cg17804611	3.21E-08	0.00331934	-0.13	IC	-25988	AK093525
**cg20295248**	**3.13E-08**	**0.00331934**	**0.27**	**IC**	**-28**	**LOC149837**
**cg21235746**	**3.07E-08**	**0.00331934**	**0.28**	**IC**	**98**	**LOC149837**
cg02363969	9.20E-08	0.00760334	-0.17	LC	360	SBK2
cg03731235	1.23E-07	0.00846603	-0.17	IC	-139576	KBTBD8
cg02790471	1.58E-07	0.00920754	-0.14	IC	79	SCML4
cg16338365	1.78E-07	0.00920754	0.20	IC	11406	GPR31
cg04875514	2.46E-07	0.00923779	-0.20	LC	-126	SOAT2
cg15906733	2.33E-07	0.00923779	-0.16	IC	32364	MIR2909
cg19630374	2.23E-07	0.00923779	-0.13	HC	1663	MYO15A
cg01619416	5.82E-07	0.01218191	-0.14	IC	-11764	SSTR5
cg05695991	4.30E-07	0.01218191	-0.19	IC	40041	LOC255512
cg07964527	5.82E-07	0.01218191	-0.15	LC	9659	PDE8A
cg08317694	3.76E-07	0.01218191	-0.15	ICshore	-521	CRABP1
cg14952449	4.90E-07	0.01218191	-0.20	LC	23722	BC032469
cg17287034	5.89E-07	0.01218191	-0.22	LC	179965	ADAM12
cg18098004	5.48E-07	0.01218191	-0.16	IC	136654	GALNT14
cg25003924	5.55E-07	0.01218191	-0.10	HC	-390	ARPP19
cg26176348	5.10E-07	0.01218191	-0.15	LC	25539	ASAP2
cg00490349	8.06E-07	0.01555485	-0.22	LC	-62	DQ593189
cg10020121	8.28E-07	0.01555485	-0.15	IC	28912	KIAA1751
cg12438037	9.11E-07	0.01584402	0.14	IC	1088	MRPS2
**cg17479830**	**9.20E-07**	**0.01584402**	**-0.17**	**IC**	**-8577**	**GALNT9**
cg06149357	9.88E-07	0.01633154	-0.20	ICshore	-715	SRD5A2
cg04978471	1.16E-06	0.01826386	0.16	LC	3248	TJAP1
cg08603125	1.28E-06	0.01826386	-0.12	LC	-1240	SRPK1
cg12618699	1.19E-06	0.01826386	-0.09	IC	3281	LOC100507055
cg19102771	1.25E-06	0.01826386	0.11	IC	-1712	CCDC140
cg10283371	1.42E-06	0.01831002	0.15	HC	18242	GADD45B
cg14698665	1.33E-06	0.01831002	0.09	LC	-3949	CTNND2
cg20424400	1.40E-06	0.01831002	-0.16	IC	14115	MVK

cg15964153	1.56E-06	0.01945317	-0.12	HC	16378	MIR4440
cg22920594	1.60E-06	0.01945317	-0.10	LC	1719	Metazoa_SRP
**cg16763089**	**1.84E-06**	**0.02117508**	**0.22**	**IC**	**-42**	**LOC149837**
cg27060386	1.82E-06	0.02117508	-0.12	ICshore	-12050	AK128777
cg11919837	2.08E-06	0.02203341	-0.24	IC	-7630	AX747062
cg17413120	2.06E-06	0.02203341	0.22	IC	17861	MMP17
cg21725652	2.05E-06	0.02203341	-0.13	LC	-25340	MIR598
cg03486485	2.23E-06	0.02246341	0.13	HC	-13253	CYP2R1
**cg10553748**	**2.18E-06**	**0.02246341**	**-0.17**	**HC**	**1695**	**CHRNE**
cg04776470	2.44E-06	0.02399467	-0.24	IC	3157	SYNJ2
cg05812599	2.82E-06	0.02709994	-0.15	ICshore	-572	CLPB
cg26071680	2.96E-06	0.02777592	0.12	HC	321	SAV1
cg03800065	3.12E-06	0.02854731	0.19	ICshore	29122	AK125849
cg12436631	3.18E-06	0.02854731	-0.15	LC	733	DCST1
cg10778379	3.38E-06	0.02971667	-0.12	IC	113	WHSC1
**cg03287299**	**3.48E-06**	**0.02993257**	**0.22**	**IC**	**-3**	**LOC149837**
cg27096232	3.69E-06	0.031141	0.24	IC	-6977	RGS12
cg06699669	3.84E-06	0.0315389	-0.16	HC	-8953	BC127192
cg21005438	3.89E-06	0.0315389	-0.09	IC	-32585	FHAD1
**cg01074305**	**4.26E-06**	**0.03199631**	**-0.15**	**IC**	**-19845**	**GALNT9**
cg06315909	4.22E-06	0.03199631	-0.10	ICshore	118	TMEM235
cg12468478	4.18E-06	0.03199631	0.09	ICshore	15	TCF21
cg21541083	4.09E-06	0.03199631	-0.13	ICshore	-778	STXBP2
cg03975922	4.36E-06	0.03221632	0.11	IC	544	ZMAT2
**cg25958766**	**4.55E-06**	**0.03298332**	**0.24**	**IC**	**-26**	**LOC149837**
cg00524676	5.26E-06	0.03744659	0.23	IC	362035	ALG10
**cg09495303**	**5.62E-06**	**0.03744659**	**-0.15**	**HC**	**1531**	**CHRNE**
cg16964102	5.47E-06	0.03744659	-0.08	IC	-7111	SPRNP1
cg21467717	5.57E-06	0.03744659	-0.11	HC	-258	KCNG2
cg27117491	5.51E-06	0.03744659	-0.09	HC	742	LOC375196
cg25350176	5.83E-06	0.03823275	-0.20	IC	-464883	SALL3
cg02799466	6.43E-06	0.04099832	-0.16	HC	-354	PID1
cg22694087	6.45E-06	0.04099832	-0.15	ICshore	19096	C3orf27
cg10298859	6.62E-06	0.04147772	-0.09	IC	121630	AK055145
cg01556799	6.81E-06	0.04183835	0.21	LC	311455	EPHA6
cg02484307	7.21E-06	0.04183835	-0.10	IC	-49688	JAZF1
cg07741910	6.92E-06	0.04183835	-0.14	LC	442	ZBTB22
cg14901751	7.25E-06	0.04183835	-0.13	IC	245370	CYP26B1
cg26068527	7.07E-06	0.04183835	-0.14	IC	-538	MIR338
**cg27009475**	**7.29E-06**	**0.04183835**	**-0.13**	**LC**	**-39536**	**EDAR**
cg26058971	7.39E-06	0.04184177	-0.19	LC	50637	SPEN
cg14133850	7.88E-06	0.04404045	-0.09	IC	1925	MPP7
cg23843407	7.99E-06	0.0440447	-0.12	LC	-19775	FJX1
cg01072786	8.21E-06	0.04465259	-0.22	HC	506	GLB1L

**cg00492108**	**9.41E-06**	**0.045386**	**-0.20**	**IC**	**54634**	**EDAR**
cg02403929	9.53E-06	0.045386	-0.09	IC	7071	RPTOR
cg10373147	9.28E-06	0.045386	0.17	LC	-156525	FSCB
cg11797145	8.73E-06	0.045386	0.10	ICshore	-315	MECR
cg15676528	8.76E-06	0.045386	-0.09	IC	-94292	YWHAQ
cg17703048	9.49E-06	0.045386	-0.14	IC	-26113	IGSF9B
cg20582976	9.55E-06	0.045386	-0.18	LC	1447	BX247991
cg22188918	8.82E-06	0.045386	-0.11	LC	-74	CLDN15
cg24696715	9.40E-06	0.045386	-0.13	IC	1411	TGFB3
cg26580609	8.68E-06	0.045386	-0.06	HC	104	NT5C1B
cg26851984	9.01E-06	0.045386	-0.20	LC	48740	C5orf48
cg07231053	1.05E-05	0.04587275	-0.14	IC	1787	GALNT2
cg13948456	1.02E-05	0.04587275	0.18	IC	-7655	GPSM1
cg17526483	1.04E-05	0.04587275	-0.24	IC	-759	EVX1
cg18804963	1.05E-05	0.04587275	0.20	LC	60371	U6
cg19769715	1.04E-05	0.04587275	-0.15	LC	-155	CAMK2A
cg21782315	1.04E-05	0.04587275	-0.12	IC	39810	LOC154449
cg23259586	1.05E-05	0.04587275	-0.19	IC	23586	AX748388
cg25745861	1.00E-05	0.04587275	-0.09	LC	-2782	TMEM54
cg13344344	1.08E-05	0.04659947	0.15	HC	7544	MAST1
cg09743654	1.12E-05	0.04676646	-0.14	HC	15047	PCBP1
cg14645926	1.12E-05	0.04676646	-0.09	LC	3415	FZD6
cg15611809	1.12E-05	0.04676646	-0.22	IC	-27750	PIK3R1
cg18635791	1.13E-05	0.04676646	0.16	LC	22479	IL5RA
cg05341384	1.19E-05	0.04715432	0.09	IC	11625	LOC146513
**cg10831901**	**1.20E-05**	**0.04715432**	**0.18**	**IC**	**-52**	**LOC149837**
cg10872844	1.19E-05	0.04715432	0.12	LC	-1404219	Mir_548
cg17657459	1.16E-05	0.04715432	0.23	LC	21069	7SK
cg19774236	1.18E-05	0.04715432	-0.16	IC	13035	GUCY2D
cg08438071	1.23E-05	0.04778108	-0.09	LC	9863	PANK1
cg21570020	1.24E-05	0.04778108	-0.08	IC	6089	NEU4
cg18638120	1.26E-05	0.04793095	-0.09	LC	2524	MRPS18A
cg25228422	1.26E-05	0.04793095	-0.29	HC	4609	TWIST1
cg02258414	1.28E-05	0.04814956	-0.16	IC	-18262	DEGS2
cg16459281	1.31E-05	0.04870058	-0.13	IC	-125226	RYBP

TSS= transcription start site, HC= high-density CpG islands (CG content >55%, Obs/Exp CpG ratio >0.75 and length >500 bps), IC = intermediate-density CpG islands (CG content >50%, Obs/Exp CpG ratio >0.48 and length >200 bps), LC= low-density CpG regions (non-islands), ICShore= regions of intermediate-density CpG island that border HCs.

S2 Table. Summary of the 849 DNA regions differentially methylated between bronchial brush isolated airway epithelial cells isolated from donors with and without asthma.

Chromosome Location	Start Location	End Location	Region Width	Number of CpGs	Minimum FDR	Max. difference in β value	Mean difference in β value	Overlapping Promoters
chr17	1083309	1083958	650	4	0.00194	0.151	0.005	ABR
chr2	162101123	162101506	384	3	1.12E-05	-0.469	-0.303	AC009299.3
chr2	220222765	220223201	437	4	0.00241	0.293	0.145	AC053503.2
chr2	233284112	233284934	823	4	0.00012	-0.288	-0.197	AC068134.5
chr15	96904912	96906732	1821	8	4.15E-08	0.439	0.252	AC087477.1
chr2	43327937	43328657	721	7	1.70E-07	0.399	0.236	AC093609.1
chr17	7982510	7982898	389	6	0.00316	0.118	0.005	ALOX12B
chr7	537126	537828	703	6	0.0001	0.359	0.009	AC147651.1
chr12	81471311	81472177	867	12	0.00029	-0.140	0.028	ACSS3
chr16	77468850	77469998	1149	8	0.0001	-0.400	-0.112	ADAMTS18
chr16	4102293	4103161	869	3	0.00142	0.267	0.205	ADCY9
chr2	100722408	100723417	1010	6	0.00133	0.260	0.153	AFF3
chr6	32151394	32151723	330	4	0.00393	0.180	0.086	AGER
chr8	141565971	141566221	251	3	0.00015	0.248	0.193	AGO2
chr2	241807747	241807924	178	3	0.00168	0.190	0.072	AGXT
chr6	31584215	31584223	9	2	0.0038	0.174	0.080	AIF1
chr17	29672336	29672644	309	2	2.46E-05	0.119	0.065	AK4P1
chr14	104178670	104179660	991	5	0.00022	0.314	0.151	AL049840.1
chr6	30580928	30582352	1425	4	7.02E-08	-0.248	-0.101	AL662800.1
chr19	6373131	6373461	331	3	0.00106	0.018	0.017	ALKBH7
chr2	190539199	190539778	580	4	0.00236	0.127	0.084	ANKAR
chr17	27920638	27920934	297	3	0.00084	-0.044	-0.021	ANKRD13B
chr15	69111367	69111511	145	3	0.00157	0.053	0.028	ANP32A
chr21	43161103	43161312	210	2	0.0005	0.226	0.112	AP001615.9

chr2	21266500	21267113	614	9	0.00194	0.364	0.124	APOB-001
chr11	116700447	116700556	110	3	0.00394	0.207	0.142	APOC3
chr4	86850721	86851425	705	5	5.88E-07	0.453	0.350	ARHGAP24
chr1	17951184	17951330	147	2	0.00203	0.190	0.177	ARHGEF10L
chr15	52861754	52861919	166	2	0.00262	0.227	0.138	ARPP19
chr14	94423801	94425114	1314	6	0.00104	0.158	0.052	ASB2
chr11	2292361	2292544	184	9	0.0036	-0.066	-0.001	ASCL2
chr13	113439513	113439949	437	3	0.00136	0.046	0.022	ATP11A
chr6	31512618	31513867	1250	10	1.38E-05	0.262	0.128	ATP6V1G2
chr17	42275334	42275458	125	2	0.0024	-0.189	-0.110	ATXN7L3
chr16	403022	403800	779	3	1.92E-07	0.136	0.057	AXIN1
chr11	134254573	134255295	723	8	0.00015	0.176	0.080	B3GAT1
chr6	91006321	91006647	327	7	0.00339	0.011	0.007	BACH2
chr8	143545940	143545949	10	2	0.0023	-0.412	-0.365	BAI1
chr17	79030539	79030796	258	2	0.00016	-0.380	-0.214	BAIAP2
chr17	79005386	79005662	277	3	0.00158	0.183	0.040	BAIAP2-AS1
chr11	129246394	129246718	325	2	1.55E-05	0.355	0.187	BARX2
chr11	129306366	129306881	516	4	0.00112	0.310	0.241	BARX2
chr2	111876752	111877177	426	3	0.00219	0.325	0.223	BCL2L11
chr1	39956653	39956807	155	3	0.00372	0.108	0.054	BMP8A
chr11	8955878	8955884	7	2	0.00065	0.180	0.093	C11orf16
chr11	64728445	64728559	115	2	0.00152	0.413	0.320	C11orf85
chr1	228401186	228402305	1120	6	0.00046	-0.199	-0.039	C1orf145
chr1	112016586	112016615	30	2	2.28E-05	0.677	0.658	C1orf162
chr21	40985387	40985406	20	2	0.00049	0.247	0.212	C21orf88
chr15	62358531	62358982	452	4	0.00251	0.061	0.017	C2CD4A
chr6	31963526	31964754	1229	7	9.43E-07	0.185	0.105	C4A-008
chr18	20714332	20715409	1078	7	0.00042	0.054	0.025	CABLES1
chr18	20735537	20735672	136	3	0.0037	0.366	0.292	CABLES1
chr3	50540137	50540261	125	3	0.00257	-0.166	-0.085	CACNA2D2

chr17	37353248	37353483	236	2	0.00175	0.031	0.021	CACNB1
chr10	18629497	18629677	181	5	0.00171	0.369	0.210	CACNB2
chr2	152954650	152954970	321	2	0.00218	0.013	0.004	CACNB4
chr11	14926738	14927004	267	3	0.00036	-0.274	-0.087	CALCB
chr7	71801793	71802574	782	3	0.00017	0.055	0.018	CALN1
chr17	76993272	76993698	427	6	2.40E-12	0.396	0.198	CANT1
chr2	241535685	241536284	600	5	3.00E-14	0.424	0.223	CAPN10
chr11	67188491	67188871	381	5	0.00184	0.014	0.012	CARNS1
chr17	73506017	73506230	214	6	0.00298	-0.275	-0.153	CASKIN2
chr16	3193279	3193410	132	2	8.61E-05	0.175	0.114	CASP16
chr19	45281236	45281526	291	4	0.00131	0.106	0.036	CBLC
chr16	49314038	49314323	286	3	0.00118	-0.323	-0.134	CBLN1
chr16	3085867	3086396	530	4	0.00133	0.173	0.074	CCDC64B
chr11	64115989	64116279	291	4	0.00298	-0.238	-0.183	CCDC88B
chr9	36169175	36169203	29	2	0.00157	0.170	0.111	CCIN
chr11	69460951	69461451	501	5	0.00016	0.153	0.103	CCND1
chr11	831170	831375	206	2	3.47E-07	0.167	0.090	CD151
chr5	66492464	66492721	258	2	4.23E-06	0.250	0.217	CD180
chr11	60869601	60869634	34	2	0.00323	0.175	0.026	CD5
chr5	27039965	27040099	135	2	0.00022	0.179	0.142	CDH9
chr11	2906934	2908036	1103	24	4.08E-05	0.222	0.035	CDKN1C
chr4	184365405	184365897	493	6	0.0023	-0.030	0.003	CDKN2AIP
chr1	22327803	22328026	224	2	0.0003	0.078	0.009	CELA3A
chr4	56813860	56813898	39	2	0.00095	0.320	0.289	CEP135
chr19	8273505	8274103	599	12	0.00053	-0.225	-0.029	CERS4
chr17	48545805	48546321	517	8	0.00046	-0.260	-0.083	CHAD
chr19	16630059	16630285	227	4	0.00382	0.174	0.105	CHERP-008, CHERP-007
chr11	882636	882687	52	3	7.11E-05	0.452	0.296	CHID1
chr8	42623686	42623946	261	6	0.00112	0.332	0.152	CHRNA6

chr19	1265302	1265458	157	2	0.002	0.032	0.003	CIRBP
chr3	170137871	170138205	335	2	0.00278	0.328	0.310	CLDN11
chr21	31587610	31588354	745	3	0.00072	0.596	0.245	CLDN8
chr1	155239301	155239493	193	4	0.00282	0.178	0.072	CLK2
chr11	123066529	123067275	747	6	9.33E-06	-0.401	-0.205	CLMP
chr13	77565875	77566922	1048	8	1.75E-05	0.024	0.010	CLN5
chr6	75911716	75912711	996	7	6.71E-15	0.384	0.288	COL12A1
chr17	48268233	48268237	5	2	0.00169	0.162	0.147	COL1A1
chr7	130353742	130354073	332	9	0.003	-0.208	-0.060	COPG2
chr7	129931729	129932033	305	3	1.83E-06	0.109	0.049	CPA4
chr16	89650950	89650991	42	2	0.00446	0.295	0.195	CPNE7
chr21	44588747	44589331	585	10	0.00038	0.163	0.070	CRYAA
chr20	54968604	54968626	23	2	0.00059	-0.302	-0.259	CSTF1
chr10	126848743	126849062	320	2	0.00052	-0.081	-0.039	CTBP2
chr5	1849983	1850261	279	2	0.00279	0.509	0.381	CTD-2194D22.1
chr5	43019660	43020451	792	6	0.00072	0.277	0.128	CTD-2201E18.3
chr17	77963988	77964703	716	4	0.00301	0.145	0.030	CTD-2529O21.1
chr16	89118603	89119709	1107	8	3.05E-06	0.490	0.355	CTD-2555A7.2
chr17	78915878	78915881	4	2	0.00325	0.202	0.113	CTD-2561B21.4
chr8	28925837	28926051	215	3	0.00063	0.164	0.085	CTD-2647L4.5
chr16	54972078	54973385	1308	9	6.37E-06	0.375	0.250	CTD-3032H12.1
chr15	22954791	22956051	1261	10	1.28E-05	0.325	0.072	CYFIP1
chr17	74533845	74534419	575	7	0.0013	0.041	0.018	CYGB-001
chr2	72371255	72371552	298	4	0.00099	0.522	0.317	CYP26B1
chr22	42538836	42538988	153	3	0.00091	0.183	0.153	CYP2D7P
chr22	42548783	42549153	371	4	0.00207	0.281	0.133	CYP2D8P
chr3	82857026	82857285	260	4	0.00077	0.166	0.090	CYP51A1P1

chr11	61106595	61106785	191	4	0.00408	0.083	0.053	DAK
chr1	155006911	155007323	413	4	7.24E-05	0.347	0.131	DCST2
chr17	76899257	76899627	371	4	4.75E-06	0.201	0.056	DDC8
chr12	57915555	57915636	82	4	0.00257	0.217	0.056	DDIT3
chr6	30851069	30851529	461	5	0.00314	0.039	0.010	DDR1
chr12	124086193	124087057	865	13	0.00021	-0.026	0.003	DDX55
chr11	46382544	46383209	666	6	4.01E-05	-0.240	-0.097	DGKZ
chr21	47878552	47878746	195	3	0.00372	0.010	0.005	DIP2A
chr20	34995278	34995517	240	4	0.00236	0.194	0.101	DLGAP4
chr2	172949481	172949501	21	2	0.00359	0.067	0.044	DLX1
chr7	96652894	96653617	724	3	0.00062	0.142	0.123	DLX5
chr5	174872330	174872371	42	2	0.00103	-0.369	-0.289	DRD1
chr2	96810531	96811264	734	5	0.00014	0.032	0.008	DUSP2
chr10	82116203	82117119	917	11	0.00171	0.222	0.022	DYDC2
chr14	102510281	102510790	510	6	1.20E-05	0.243	0.044	DYNC1H1
chr20	57875318	57875545	228	7	0.00433	0.103	0.006	EDN3
chr12	107296891	107297296	406	5	0.00202	0.251	0.099	EEF1B2P4
chr1	35586358	35586649	292	5	0.00118	-0.299	-0.223	EFCAB14P1
chr14	23834860	23835595	736	8	0.00114	0.036	0.003	EFS
chr6	32135070	32135396	327	13	0.00331	0.261	0.071	EGFL8
chr8	22548546	22548952	407	3	0.00089	-0.271	-0.193	EGR3
chr6	31854757	31856329	1573	26	2.39E-06	-0.135	0.026	EHMT2
chr17	7475727	7476167	441	11	0.00211	0.044	-0.005	EIF4A1
chr1	79471984	79472917	934	11	1.36E-07	0.354	0.175	ELTD1
chr3	125567161	125567258	98	3	0.00155	0.146	0.065	ENPP7P4
chr6	152127812	152127887	76	3	0.00104	0.181	0.078	ESR1
chr17	74005445	74005651	207	2	0.00232	0.116	0.097	EVPL
chr7	27280585	27281964	1380	13	3.83E-08	0.373	0.212	EVX1
chr12	111799994	111801411	1418	8	0.00035	0.122	0.054	FAM109A
chr13	46189838	46190198	361	8	0.00188	-0.163	-0.031	FAM194B

chr21	46358532	46358702	171	2	0.00018	0.413	0.382	FAM207A
chr10	88779109	88779941	833	7	0.00011	0.128	0.089	FAM25A
chr6	3848218	3850106	1889	32	3.34E-25	-0.254	0.136	FAM50B
chr19	55873515	55873983	469	4	0.00265	0.191	0.062	FAM71E2
chr12	133066061	133066762	702	17	0.0022	0.018	0.005	FBRSL1
chr1	11710460	11711141	682	3	7.47E-05	0.358	0.224	FBXO2-004
chr8	38323238	38323291	54	2	0.00063	0.366	0.305	FGFR1
chr10	123353298	123354171	874	13	3.63E-12	0.303	0.222	FGFR2
chr16	67282407	67282837	431	3	0.00322	0.011	0.010	FHOD1
chr12	2903563	2904419	857	10	0.00063	0.024	0.004	FKBP4
chr7	128471146	128471600	455	2	0.00276	-0.161	-0.154	FLNC
chr17	80673184	80673675	492	2	0.00015	0.232	0.180	FN3KRP
chr6	1607922	1608977	1056	8	6.93E-06	0.440	0.198	FOXC1
chr14	29236898	29237480	583	3	0.0013	0.162	0.118	FOXG1
chr10	14051636	14052028	393	6	0.00178	0.210	0.170	FRMD4A
chr2	181738051	181738271	221	3	0.00436	0.195	0.169	FTH1P20
chr19	35606534	35607221	688	6	0.00017	0.050	0.024	FXYD3
chr19	35633669	35633794	126	2	0.00448	0.014	0.012	FXYD7
chr5	39219245	39220149	905	4	0.00038	0.119	0.070	FYB
chr2	202901045	202901470	426	5	6.54E-07	0.459	0.345	FZD7
chr7	72847175	72848481	1307	6	9.61E-05	-0.204	-0.010	FZD9
chr15	27111884	27112430	547	11	0.00159	-0.228	-0.041	GABRA5
chr2	242716388	242716801	414	4	0.00169	-0.280	-0.199	GAL3ST2
chr3	16216094	16216215	122	4	0.00186	0.122	0.088	GALNT15
chr10	8095960	8098328	2369	30	1.22E-07	-0.310	0.004	GATA3
chr10	8100114	8101566	1453	7	1.16E-08	-0.367	-0.179	GATA3
chr2	237075188	237075576	389	2	0.00025	0.040	0.033	GBX2
chr6	10529972	10530035	64	2	0.00034	0.436	0.435	GCNT2
chr20	25387977	25388289	313	6	0.00438	-0.128	-0.020	GINS1
chr1	35245692	35245996	305	3	9.64E-05	0.226	0.096	GJB3

chr15	35046610	35046765	156	3	0.00042	0.239	0.144	GJD2
chr8	144358043	144358898	856	3	0.00024	0.167	0.143	GLI4
chr16	4364093	4364150	58	2	0.00399	-0.158	-0.019	GLIS2
chr6	1624386	1624720	335	3	8.75E-06	0.326	0.174	GMDS
chr20	57415144	57415697	554	4	0.00088	0.156	0.071	GNAS
chr16	850562	851298	737	7	0.00035	0.143	0.033	GNG13
chr1	235813839	235814462	624	11	0.00085	0.240	0.043	GNG4
chr3	128778738	128780164	1427	9	0.00011	0.208	0.067	GP9
chr1	27216367	27216933	567	8	0.00192	-0.038	-0.003	GPN2
chr6	46889624	46890512	889	5	1.83E-06	-0.399	-0.156	GPR116
chr6	32164801	32165200	400	4	0.0028	0.317	0.192	GPSM3
chr17	37895424	37896620	1197	7	6.09E-06	0.071	0.016	GRB7
chr6	101846967	101847706	740	8	7.08E-05	0.381	0.223	GRIK2
chr13	21893289	21893662	374	4	0.00063	0.169	0.066	GRK6P1
chr13	114018021	114018173	153	2	0.00159	0.019	0.008	GRTP1
chr14	95236123	95236615	493	5	0.0033	-0.138	-0.056	GSC
chr6	30878952	30879000	49	2	0.00403	0.144	0.076	GTF2H4
chr7	73867797	73868870	1074	11	0.00141	-0.043	-0.001	GTF2IRD1
chr11	2020417	2020560	144	4	0.00239	0.081	0.049	H19
chr1	226249809	226250443	635	9	0.00308	-0.028	0.000	H3F3A
chr10	115312553	115312600	48	2	0.00044	0.260	0.167	HABP2
chr19	35772657	35772917	261	2	0.00255	0.310	0.121	HAMP
chr16	222837	223408	572	4	6.38E-05	-0.125	-0.045	HBA2
chr6	29974022	29975154	1133	32	9.57E-05	0.099	0.003	HCG4P3
chr2	239988369	239988451	83	2	0.00125	0.234	0.140	HDAC4
chr11	93754094	93754390	297	4	0.00077	0.108	0.072	HEPHL1
chr17	8028735	8028948	214	4	0.00264	0.052	0.015	HES7
chr4	3442962	3444147	1186	9	1.07E-05	0.205	0.084	HGFAC
chr17	79663964	79664552	589	4	2.59E-05	0.189	0.098	HGS
chr12	51346947	51347099	153	3	0.00235	0.180	0.140	HIGD1C

chr6	26225520	26226256	737	5	0.00041	0.342	0.144	HIST1H3E
chr6	26239995	26240579	585	6	4.78E-06	0.147	0.062	HIST1H4F
chr1	228644750	228645710	961	17	5.93E-09	0.275	0.053	HIST3H2A
chr1	228612603	228613260	658	10	0.00044	0.119	0.043	HIST3H3
chr1	42503579	42503768	190	2	0.00099	0.372	0.163	HIVEP3
chr6	32920567	32920880	314	5	0.00259	0.237	0.079	HLA-DMA
chr6	29855110	29856141	1032	13	0.00156	0.109	0.013	HLA-H-201
chr12	10264389	10265096	708	3	0.00045	0.191	0.055	HNRNPABP1
chr7	27180888	27181067	180	2	0.00158	-0.342	-0.110	HOXA5
chr12	54333179	54333224	46	2	0.00275	-0.239	-0.135	HOXC13
chr12	54446019	54446576	558	7	1.44E-05	-0.341	-0.198	HOXC4
chr2	129076914	129077232	319	2	0.00143	0.275	0.127	HS6ST1
chr14	65005951	65006359	409	9	1.14E-06	0.333	0.093	HSPA2
chr4	3231575	3231885	311	3	0.002	0.168	0.099	HTT
chr21	33247276	33248032	757	3	0.00071	0.639	0.309	HUNK
chr1	228362309	228362509	201	3	0.00246	0.311	0.229	IBA57
chr1	6284844	6285107	264	3	0.00358	0.106	0.070	ICMT
chr10	1068490	1068567	78	3	0.00396	-0.315	-0.225	IDI2-AS1
chr6	30710200	30711644	1445	35	0.00112	-0.042	0.001	IER3
chr1	19280186	19280546	361	2	2.04E-06	0.391	0.200	IFFO2
chr17	47074576	47075880	1305	11	0.00024	0.271	0.051	IGF2BP1
chr16	1842068	1842505	438	3	0.00398	0.182	0.035	IGFALS
chr1	159916077	159916472	396	2	0.00026	0.266	0.142	IGSF9
chr19	18197544	18197853	310	4	0.00212	-0.337	-0.189	IL12RB1
chr20	20347495	20347593	99	2	3.35E-06	0.292	0.133	INSM1
chr5	39721549	39721760	212	4	0.0033	-0.104	0.018	INTS6P1
chr6	154677796	154678326	531	3	1.88E-05	0.177	0.035	IPCEF1
chr5	75701065	75701460	396	2	0.00058	0.191	0.160	IQGAP2
chr1	234746099	234746800	702	8	0.00156	-0.017	-0.002	IRF2BP2
chr11	616879	617708	830	6	0.00011	0.136	0.042	IRF7

chr5	2753746	2756361	2616	18	8.86E-06	0.499	0.231	IRX2
chr1	949392	949893	502	5	4.87E-06	0.249	0.129	ISG15
chr15	76628154	76629110	957	8	1.23E-05	0.294	0.091	ISL2
chr15	74422545	74423449	905	11	9.89E-05	-0.164	-0.025	ISLR2
chr19	55965878	55966376	499	2	0.00115	0.214	0.163	ISOC2
chr21	46350872	46351329	458	3	0.00036	-0.198	-0.149	ITGB2
chr15	41794412	41794747	336	2	0.00037	0.227	0.189	ITPKA
chr11	133937475	133937493	19	2	0.00445	0.341	0.308	JAM3
chr22	39096679	39097540	862	5	4.57E-05	-0.027	0.001	JOSD1
chr3	124103560	124103696	137	4	0.00048	0.481	0.462	KALRN
chr19	49575475	49575664	190	2	0.00363	0.152	0.103	KCNA7
chr3	155838109	155838625	517	4	2.00E-07	0.367	0.230	KCNAB1
chr11	2883529	2884145	617	7	0.00041	0.143	0.006	KCNQ1-AS1
chr6	73329988	73330358	371	5	9.44E-05	0.311	0.289	KCNQ5
chr8	99440279	99440522	244	3	0.00045	0.258	0.168	KCNS2
chr6	168416740	168417743	1004	7	3.03E-11	0.488	0.371	KIF25
chr15	90199883	90200115	233	2	2.02E-05	0.197	0.116	KIF7
chr15	31619949	31620290	342	2	0.00017	0.021	0.011	KLF13
chr10	3828932	3829456	525	2	0.00055	0.346	0.306	KLF6
chr22	50986031	50987294	1264	6	6.03E-05	-0.374	-0.255	KLHDC7B
chr1	161068138	161068715	578	4	0.00324	-0.024	-0.010	KLHDC9
chr19	51486901	51487136	236	3	0.00184	0.282	0.141	KLK7
chr17	39742965	39743427	463	3	0.00089	0.439	0.214	KRT14
chr12	52827847	52829316	1470	8	4.00E-07	0.213	0.143	KRT75
chr12	52586635	52586810	176	2	0.00313	0.221	0.156	KRT80
chr12	52714864	52715691	828	5	5.54E-06	0.315	0.115	KRT83
chr12	52760975	52762005	1031	7	0.00015	0.181	0.030	KRT85
chr21	46077454	46078007	554	6	0.00012	0.148	0.073	KRTAP12
chr11	1717919	1718586	668	3	3.70E-05	0.153	0.127	KRTAP5-6

chr16	1682412	1682446	35	2	0.00012	0.336	0.225	LA16c-431H6.6
chr1	201368390	201369650	1261	11	1.10E-05	-0.115	-0.003	LAD1-002, LAD1-001
chr12	6881118	6882083	966	9	3.47E-07	-0.358	-0.163	LAG3
chr16	28995994	28996533	540	11	0.00055	0.220	0.105	LAT
chr20	36974764	36975083	320	5	2.26E-05	0.163	0.062	LBP
chr2	74731354	74731413	60	3	0.00092	0.310	0.200	LBX2
chr9	98782756	98782979	224	2	0.00136	0.303	0.103	LINC00092
chr13	24040391	24040510	120	2	0.00175	0.061	0.008	LINC00327
chr5	87967728	87968528	801	5	3.02E-06	0.245	0.058	LINC00461
chr4	124694137	124694178	42	2	0.00018	0.241	0.234	LINC01091
chr2	71115787	71116411	625	4	0.00067	0.123	0.058	LINC01143
chr4	54457645	54458246	602	6	0.00051	0.155	0.057	LNX1
chr17	66194137	66195485	1349	14	4.26E-09	0.317	0.121	LOC440461
chr19	14273676	14274172	497	4	7.87E-05	0.139	0.059	LPHN1-004
chr1	53794313	53795111	799	3	4.09E-07	0.238	0.166	LRP8
chr6	31548843	31550090	1248	18	3.28E-05	0.205	0.033	LTB
chr11	65306116	65306186	71	2	0.00063	-0.185	-0.085	LTBP3
chr1	23496304	23496648	345	2	0.00035	0.260	0.233	LUZP1
chr6	31650735	31651362	628	17	0.00018	0.222	-0.048	LY6G5C
chr22	21335304	21335632	329	2	0.00064	0.221	0.132	LZTR1
chr13	36049807	36051073	1267	10	0.00031	0.448	0.315	MAB21L1
chr1	11751781	11753338	1558	7	2.24E-07	-0.319	-0.038	MAD2L2
chr7	1570208	1570663	456	5	0.00039	0.032	0.016	MAFK
chr5	71475111	71475356	246	3	1.88E-05	0.257	0.130	MAP1B
chr11	65374955	65375203	249	4	0.00207	0.243	0.153	MAP3K11
chr21	30451398	30452272	875	3	1.78E-09	0.233	0.115	MAP3K7CL
chr14	71250264	71250424	161	3	0.00029	0.387	0.208	MAP3K9
chr11	45923319	45924191	873	2	0.00037	0.152	0.142	MAPK8IP1
chr2	27239543	27239739	197	2	0.00059	0.185	0.103	MAPRE3

chr17	43971177	43971471	295	3	0.00238	-0.116	-0.079	MAPT
chr13	113556413	113556486	74	2	0.00031	0.530	0.362	MCF2L-015, MCF2L-016, MCF2L-205
chr2	131010075	131010871	797	6	6.66E-05	0.401	0.224	MED15P5
chr14	101291136	101292306	1171	19	0.00044	0.235	0.069	MEG3
chr7	130130918	130131268	351	7	0.00336	0.047	0.034	MEST
chr4	2935692	2936284	593	8	0.00042	-0.030	0.004	MFSD10
chr17	74733438	74733682	245	7	0.00439	-0.045	-0.002	MFSD11
chr16	4714443	4714815	373	5	2.22E-05	0.316	0.146	MGRN1
chr22	18325160	18325505	346	2	0.00115	0.334	0.328	MICAL3
chr6	31465865	31466187	323	10	0.00201	-0.017	0.005	MICB
chr17	19247837	19248565	729	9	0.00016	-0.371	-0.181	MIR1180
chr22	45596797	45597767	971	10	6.46E-06	0.299	0.181	MIR1249
chr15	86315115	86315211	97	3	6.96E-06	0.390	0.309	MIR1276
chr15	86313046	86313973	928	8	0.00012	0.294	0.180	MIR1276
chr21	26934197	26934455	259	3	0.00181	0.103	0.046	MIR155HG
chr20	57393844	57394097	254	3	0.00013	0.278	0.220	MIR296
chr8	96085270	96085448	179	3	0.00364	0.341	0.251	MIR3150A
chr16	67236231	67236337	107	2	0.00331	0.084	0.073	MIR328
chr1	9212514	9212754	241	3	0.0004	0.164	0.116	MIR34A
chr10	118925704	118925818	115	2	0.00194	-0.103	-0.043	MIR3663
chr4	2251938	2252007	70	2	0.00386	-0.208	-0.185	MIR4800
chr12	95226786	95226993	208	3	7.63E-07	0.349	0.224	MIR492
chr9	125874627	125874949	323	2	0.00074	-0.275	-0.138	MIR600HG
chr19	3962150	3962194	45	2	0.00309	0.255	0.178	MIR637
chr17	56296444	56297515	1072	15	1.03E-13	0.503	0.129	MKS1
chr22	50523686	50524691	1006	13	3.15E-05	0.335	0.086	MLC1
chr8	144653570	144653975	406	5	0.00019	0.040	0.008	MROH6
chr9	138393270	138393570	301	2	1.07E-05	-0.180	-0.080	MRPS2
chr14	105932930	105933283	354	3	0.00029	0.194	0.059	MTA1

chr17	48585216	48585270	55	2	0.00264	-0.256	-0.218	MYCBPAP
chr6	1615236	1617619	2384	8	2.93E-16	0.720	0.493	NA
chr14	104979372	104980775	1404	6	2.24E-18	0.562	0.444	NA
chr11	115581830	115581955	126	3	3.24E-12	0.685	0.514	NA
chr3	140813919	140814133	215	3	1.34E-15	0.581	0.527	NA
chr8	1906338	1906498	161	3	1.28E-11	0.459	0.436	NA
chr6	101849938	101850881	944	3	5.38E-07	0.461	0.336	NA
chr6	164345272	164345307	36	2	2.29E-06	0.401	0.333	NA
chr20	58983676	58983939	264	2	3.17E-06	0.389	0.251	NA
chr5	72607540	72607795	256	2	1.62E-05	0.517	0.476	NA
chr20	20433168	20433328	161	3	2.22E-07	0.413	0.326	NA
chr19	872690	873330	641	2	4.43E-06	-0.401	-0.386	NA
chr8	102121398	102121621	224	2	1.01E-05	0.593	0.506	NA
chr12	5537882	5538415	534	2	0.00031	-0.272	-0.229	NA
chr12	117042854	117042917	64	3	5.20E-05	0.628	0.564	NA
chr8	67419572	67419936	365	2	0.00029	0.305	0.285	NA
chr19	33892576	33892662	87	2	0.00038	0.162	0.142	NA
chr5	3932145	3932233	89	2	0.00037	0.577	0.459	NA
chr2	128453108	128453484	377	5	2.04E-09	0.423	0.352	NA
chr8	143473852	143473903	52	2	0.00018	0.136	0.092	NA
chr6	138785358	138785451	94	2	0.00053	0.135	0.126	NA
chr5	1973774	1974131	358	3	4.56E-05	0.468	0.257	NA
chr19	13208349	13208599	251	2	0.00015	-0.271	-0.238	NA
chr7	90895894	90896701	808	4	1.27E-06	0.528	0.365	NA
chr7	156309329	156309358	30	2	0.00155	0.315	0.265	NA
chr17	840950	840975	26	2	0.00246	0.248	0.209	NA
chr19	42880974	42881132	159	2	0.0003	0.160	0.134	NA
chr4	11652441	11652505	65	3	1.07E-05	0.493	0.384	NA
chr8	1895431	1895558	128	2	0.00023	0.275	0.274	NA
chr10	521758	521830	73	3	0.00013	0.306	0.289	NA

chr10	134221633	134221855	223	2	3.20E-05	0.042	0.040	NA
chr6	99236007	99236061	55	2	0.00034	0.285	0.196	NA
chr19	49061546	49061702	157	3	0.00012	0.426	0.357	NA
chr16	86860774	86860801	28	2	0.00265	0.160	0.159	NA
chr11	122848983	122849037	55	2	0.00119	-0.253	-0.221	NA
chr9	98491087	98491311	225	2	0.00098	0.225	0.224	NA
chr12	130921499	130921861	363	2	0.00241	0.142	0.137	NA
chr7	632926	633202	277	3	3.09E-05	0.496	0.386	NA
chr5	170877540	170878246	707	5	5.36E-07	-0.478	-0.387	NA
chr15	45409170	45409319	150	2	0.00274	0.273	0.228	NA
chr1	2188767	2188894	128	2	0.00127	0.367	0.311	NA
chr14	102918905	102918977	73	2	0.00131	0.212	0.189	NA
chr17	80943851	80944193	343	3	0.00025	0.301	0.232	NA
chr1	37337827	37337877	51	2	0.00046	0.152	0.111	NA
chr12	127384772	127384868	97	2	0.00154	0.184	0.184	NA
chr5	76940356	76940449	94	2	0.00071	0.355	0.346	NA
chr7	630473	630914	442	5	6.94E-08	0.421	0.335	NA
chr7	73720808	73721031	224	3	0.00012	0.383	0.286	NA
chr1	10757892	10758237	346	2	0.00046	0.333	0.159	NA
chr16	86994949	86995081	133	3	0.00011	0.381	0.317	NA
chr2	101008252	101008576	325	3	8.41E-06	0.259	0.243	NA
chr17	79400831	79400976	146	3	0.00029	0.179	0.105	NA
chr3	11519599	11519911	313	3	0.00013	0.167	0.159	NA
chr2	9865398	9865477	80	2	0.00063	0.179	0.174	NA
chr9	133794519	133794590	72	2	0.00422	0.251	0.223	NA
chr2	101434082	101434611	530	2	0.00178	0.269	0.176	NA
chr1	215993017	215993110	94	2	0.003	0.194	0.172	NA
chr16	51026724	51026841	118	3	0.00039	0.453	0.396	NA
chr1	228131966	228131977	12	2	0.00296	0.394	0.279	NA
chr19	30935791	30936625	835	3	0.00054	0.136	0.082	NA

chr6	7142024	7142066	43	2	0.00481	0.385	0.306	NA
chr7	1949632	1949824	193	2	0.00077	0.221	0.148	NA
chr2	54936739	54936822	84	2	0.00339	-0.196	-0.175	NA
chr10	133048362	133048392	31	2	0.00083	-0.252	-0.246	NA
chr11	8385712	8385767	56	2	0.00413	0.479	0.380	NA
chr16	88935317	88935474	158	2	0.00059	0.547	0.366	NA
chr1	3017674	3017794	121	2	0.00308	0.132	0.125	NA
chr1	2792637	2792744	108	3	0.00092	-0.252	-0.237	NA
chr6	1618814	1620137	1324	7	1.88E-09	0.493	0.340	NA
chr5	2562302	2562830	529	2	0.0002	0.461	0.290	NA
chr9	133413080	133413095	16	2	0.00099	0.383	0.280	NA
chr3	177494108	177494197	90	2	0.00313	0.436	0.268	NA
chr1	3289798	3289915	118	2	4.23E-05	0.235	0.186	NA
chr6	106957404	106957497	94	2	0.00151	0.140	0.136	NA
chr9	14350039	14350791	753	2	0.00184	0.489	0.397	NA
chr16	31487813	31488238	426	3	0.00097	-0.309	-0.295	NA
chr14	104741448	104742172	725	4	4.96E-06	0.379	0.257	NA
chr1	27028974	27029290	317	2	0.0008	0.053	0.037	NA
chr7	2239803	2240376	574	3	0.00031	0.060	0.025	NA
chr2	3473852	3474169	318	2	0.00323	0.358	0.329	NA
chr8	1707477	1707553	77	2	0.00403	0.247	0.208	NA
chr17	77767084	77767242	159	2	0.00399	0.247	0.172	NA
chr10	1260004	1260230	227	2	0.00262	0.129	0.101	NA
chr1	3809431	3809532	102	2	0.00027	0.458	0.317	NA
chr11	2603221	2603429	209	2	0.00349	0.052	0.050	NA
chr22	46472568	46473721	1154	5	6.12E-05	0.307	0.215	NA
chr7	57071509	57071676	168	2	0.00294	-0.299	-0.101	NA
chr1	27687685	27687695	11	2	0.00333	0.124	0.117	NA
chr17	77669181	77669222	42	3	0.00338	0.272	0.197	NA
chr14	91738961	91739219	259	4	4.53E-06	0.441	0.291	NA

chr7	788332	790384	2053	6	1.49E-05	0.582	0.162	NA
chr10	30317625	30317994	370	3	2.32E-05	0.494	0.334	NA
chr7	88315867	88315898	32	2	0.00391	0.116	0.091	NA
chr1	3115085	3115545	461	4	1.31E-05	0.484	0.359	NA
chr10	411741	411837	97	3	0.00232	0.143	0.131	NA
chr15	96884949	96892116	7168	33	6.90E-17	0.428	0.234	NA
chr1	3010896	3011304	409	3	1.79E-05	0.130	0.127	NA
chr7	139164675	139164778	104	2	0.00308	0.313	0.076	NA
chr2	240196078	240196284	207	3	0.00147	0.287	0.276	NA
chr17	78039096	78039528	433	4	0.00053	0.307	0.274	NA
chr7	668292	668612	321	2	0.00077	0.136	0.056	NA
chr16	857454	857981	528	4	0.00067	0.267	0.235	NA
chr10	131514248	131514331	84	2	9.65E-06	-0.494	-0.361	NA
chr10	131697080	131697345	266	3	0.00168	-0.321	-0.267	NA
chr3	156837590	156838403	814	6	2.43E-14	-0.540	-0.284	NA
chr10	134821734	134822001	268	3	0.00245	0.111	0.108	NA
chr11	2233644	2234043	400	2	0.00083	-0.317	-0.139	NA
chr5	1016279	1016768	490	5	4.71E-07	0.225	0.156	NA
chr4	7301491	7301823	333	3	0.00122	0.165	0.121	NA
chr5	92684137	92684412	276	4	0.0022	0.396	0.306	NA
chr16	88512529	88512653	125	2	0.00116	-0.259	-0.089	NA
chr10	1156113	1156594	482	6	2.39E-06	0.333	0.173	NA
chr7	2250050	2250274	225	2	0.00023	0.159	0.153	NA
chr5	2442958	2443073	116	3	0.00349	0.295	0.184	NA
chr21	46306688	46306794	107	3	0.00068	0.140	0.096	NA
chr11	126286370	126287023	654	5	0.00057	-0.240	-0.211	NA
chr19	49699879	49700427	549	4	0.00233	0.272	0.209	NA
chr8	21964427	21964567	141	2	0.00107	0.206	0.149	NA
chr3	13060741	13060974	234	4	0.00058	0.211	0.151	NA
chr7	5258357	5258485	129	3	0.00017	0.202	0.087	NA

chr1	15655764	15656086	323	3	0.00064	0.307	0.209	NA
chr7	1864317	1864451	135	2	0.0016	0.190	0.138	NA
chr14	102990211	102990501	291	3	0.0002	-0.289	-0.159	NA
chr2	240032685	240033412	728	5	2.31E-05	0.161	0.084	NA
chr18	77218862	77219495	634	3	0.00036	0.287	0.160	NA
chr1	15541446	15541956	511	3	0.00315	0.467	0.202	NA
chr16	85671191	85671771	581	4	0.00016	-0.221	-0.167	NA
chr4	1029410	1029654	245	4	0.00021	0.304	0.224	NA
chr10	134829832	134830157	326	2	0.00188	0.191	0.042	NA
chr1	3379492	3379884	393	3	1.93E-05	0.192	0.109	NA
chr1	91190891	91191232	342	4	0.00339	0.199	0.116	NA
chr5	501343	502920	1578	7	3.09E-07	0.441	0.307	NA
chr5	1054113	1054262	150	2	0.00341	0.126	0.085	NA
chr16	88482626	88482940	315	3	0.00193	0.178	0.120	NA
chr2	177036436	177036809	374	2	0.00088	0.177	0.076	NA
chr17	1478889	1479213	325	2	0.00354	0.361	0.265	NA
chr6	168811070	168811287	218	3	0.00018	0.426	0.299	NA
chr7	1233285	1233469	185	2	0.00059	-0.265	-0.065	NA
chr17	80827952	80827971	20	2	0.00272	0.072	0.045	NA
chr13	112905729	112905775	47	3	0.00154	-0.078	-0.044	NA
chr4	2275801	2276044	244	5	0.00021	0.202	0.136	NA
chr14	95330880	95331466	587	5	1.05E-08	-0.329	-0.227	NA
chr7	21209338	21209781	444	4	0.00098	0.401	0.288	NA
chr4	7373022	7373451	430	5	0.00052	-0.403	-0.246	NA
chr1	41978691	41979124	434	5	9.36E-08	0.224	0.120	NA
chr1	16318931	16318994	64	2	0.00063	0.121	0.101	NA
chr17	79412583	79412788	206	4	0.00343	0.181	0.059	NA
chr22	43058692	43059020	329	2	0.00028	-0.230	-0.092	NA
chr10	133743048	133743453	406	3	0.00029	0.283	0.049	NA
chr11	64146487	64146871	385	4	0.00155	0.376	0.224	NA

chr15	98937633	98937852	220	2	0.00273	0.382	0.220	NA
chr5	510035	510421	387	3	0.00083	0.425	0.261	NA
chr6	41528345	41529269	925	8	1.75E-07	0.463	0.341	NA
chr19	16643451	16644066	616	3	3.29E-05	-0.148	0.008	NA
chr1	9078214	9078477	264	3	0.00094	0.222	0.161	NA
chr2	121970401	121970600	200	3	0.00055	0.181	0.081	NA
chr21	27372387	27372461	75	4	0.00231	0.151	0.106	NA
chr17	72839038	72839750	713	3	5.23E-05	0.230	0.102	NA
chr15	96952113	96952425	313	4	8.78E-05	-0.355	-0.199	NA
chr14	105676791	105677543	753	4	1.13E-05	0.216	0.109	NA
chr6	110721138	110721629	492	4	0.00262	-0.205	-0.097	NA
chr20	44978848	44979100	253	2	0.0022	0.126	0.108	NA
chr22	30125353	30125483	131	2	0.00265	0.161	0.078	NA
chr12	30976867	30976950	84	2	0.00117	0.301	0.156	NA
chr15	53075877	53076235	359	2	2.68E-05	0.183	0.106	NA
chr7	4786364	4786986	623	6	0.00017	0.239	0.139	NA
chr13	68682018	68682777	760	3	0.00092	0.202	0.122	NA
chr2	3691145	3691651	507	6	0.00065	0.184	0.090	NA
chr6	33272880	33273014	135	4	0.00064	0.260	0.168	NA
chr3	113417997	113418063	67	3	0.0028	0.192	0.073	NA
chr1	2221807	2222674	868	5	0.00032	0.225	0.132	NA
chr7	155261034	155261402	369	4	0.00045	0.214	0.087	NA
chr6	44528765	44529157	393	5	9.90E-07	0.328	0.206	NA
chr2	66804865	66805939	1075	6	5.65E-05	0.346	0.159	NA
chr6	106582638	106583218	581	5	2.64E-08	0.338	0.180	NA
chr1	2999196	2999728	533	4	0.00033	0.231	0.129	NA
chr3	11600100	11600407	308	2	0.00032	0.239	0.162	NA
chr6	166422626	166422741	116	3	0.00215	0.160	0.068	NA
chr1	85721250	85721327	78	2	0.00436	0.174	0.034	NA
chr13	111061780	111062287	508	5	0.00059	0.222	0.128	NA

chr5	126564606	126565042	437	4	0.00018	0.257	0.122	NA
chr10	532287	532908	622	5	0.00011	0.273	0.147	NA
chr4	155253864	155254602	739	6	6.94E-08	-0.462	-0.255	NA
chr7	113722758	113723786	1029	7	4.36E-11	0.556	0.378	NA
chr3	176862885	176862949	65	2	0.00407	0.143	0.061	NA
chr21	45575014	45575832	819	4	0.0001	0.318	0.159	NA
chr2	121523609	121523682	74	3	0.0016	0.179	0.110	NA
chr2	105853494	105853672	179	3	0.00333	-0.298	-0.218	NA
chr10	105362388	105362799	412	4	0.00063	0.197	0.042	NA
chr1	1062237	1063186	950	3	0.00038	0.231	0.119	NA
chr4	4856820	4857441	622	5	0.0017	0.254	0.162	NA
chr5	158478533	158478734	202	3	0.00058	0.150	0.008	NA
chr17	78865087	78866579	1493	9	4.68E-07	0.384	0.274	NA
chr15	100533202	100533336	135	4	0.00375	0.182	0.105	NA
chr2	128185947	128186748	802	5	1.07E-05	0.117	0.060	NA
chr3	12856650	12856862	213	4	0.00382	0.148	0.054	NA
chr12	54089240	54089486	247	3	0.00114	0.230	0.089	NA
chr1	3352257	3352544	288	3	0.00267	0.131	0.093	NA
chr20	3730680	3730943	264	2	0.00213	0.049	0.047	NA
chr16	84336042	84336445	404	4	0.00039	0.484	0.274	NA
chr6	29987762	29988222	461	4	0.00141	0.212	0.095	NA
chr7	1034817	1035418	602	5	7.30E-08	0.199	0.074	NA
chr11	1483099	1483169	71	2	0.00349	-0.208	-0.097	NA
chr6	29980634	29980960	327	2	2.82E-06	0.182	0.106	NA
chr3	11597868	11597941	74	3	0.00138	0.302	0.198	NA
chr16	90002255	90002892	638	5	1.97E-05	0.194	0.128	NA
chr22	37603155	37603819	665	4	0.00037	0.194	0.095	NA
chr2	242713790	242714091	302	4	0.00045	-0.386	-0.159	NA
chr6	156718177	156718546	370	5	0.0023	0.361	0.198	NA
chr7	155276437	155276880	444	5	0.00011	0.312	0.168	NA

chr6	37673335	37673439	105	2	0.00483	0.022	0.013	NA
chr12	131237654	131237708	55	3	0.00373	-0.260	-0.152	NA
chr1	2827410	2828293	884	8	7.00E-10	0.367	0.217	NA
chr8	1310380	1310451	72	2	0.00079	0.190	0.112	NA
chr19	7541760	7542191	432	4	1.06E-05	0.278	0.112	NA
chr5	42994709	42995527	819	7	2.02E-05	0.185	0.085	NA
chr7	73894884	73895278	395	5	0.00323	-0.043	0.009	NA
chr8	26047780	26048414	635	7	0.00063	0.173	0.088	NA
chr14	99613193	99613313	121	3	0.00336	0.177	0.030	NA
chr5	2743124	2743655	532	5	0.00037	0.188	0.083	NA
chr15	70326224	70326383	160	3	0.00273	0.387	0.201	NA
chr10	407721	407935	215	3	0.00343	0.244	0.002	NA
chr2	66809780	66810606	827	8	7.24E-05	0.354	0.170	NA
chr12	121687747	121688459	713	5	0.00039	0.191	0.110	NA
chr7	65878352	65878976	625	5	0.00264	0.416	0.097	NA
chr10	125033770	125034002	233	4	0.00178	-0.304	-0.172	NA
chr19	17439208	17439339	132	2	0.00289	0.208	0.096	NA
chr15	72581569	72582316	748	4	0.00121	0.229	0.131	NA
chr4	1558451	1559244	794	6	2.16E-05	0.386	0.068	NA
chr7	55224743	55225183	441	6	0.00331	0.207	0.068	NA
chr16	898973	899108	136	3	0.00115	0.143	0.088	NA
chr12	132680733	132680972	240	3	0.00053	0.281	0.164	NA
chr8	143192949	143193131	183	3	0.00105	0.177	0.113	NA
chr16	85478769	85479301	533	6	0.00051	0.220	0.064	NA
chr10	114610560	114610803	244	4	1.59E-08	0.153	0.100	NA
chr17	80137075	80137377	303	3	0.00275	0.186	0.081	NA
chr18	77193464	77193759	296	4	5.11E-05	0.079	0.056	NA
chr2	172972681	172974819	2139	10	0.00194	0.461	-0.036	NA
chr17	46795860	46797042	1183	6	0.00012	0.341	0.130	NA
chr20	61590751	61591209	459	4	0.00143	0.252	0.065	NA

chr8	142179697	142180543	847	5	2.57E-05	-0.127	-0.003	NA
chr7	16438022	16438851	830	7	0.0021	0.089	0.018	NA
chr6	28446794	28447115	322	5	0.00332	0.242	0.157	NA
chr16	46461209	46461571	363	2	0.00093	-0.292	-0.149	NA
chr2	198029141	198029332	192	3	0.00127	-0.321	-0.215	NA
chr5	1154848	1154998	151	3	9.43E-07	0.297	0.056	NA
chr5	122421195	122421508	314	2	0.00018	0.318	0.161	NA
chr12	66122497	66123496	1000	6	0.00012	0.363	0.167	NA
chr11	59317890	59318534	645	5	0.00097	-0.113	-0.042	NA
chr17	78795392	78795669	278	3	0.00139	0.191	0.045	NA
chr19	48956129	48956265	137	3	0.00039	0.065	0.036	NA
chr11	132948586	132949126	541	3	0.0011	0.184	0.047	NA
chr10	102986306	102986686	381	5	0.00248	0.061	0.016	NA
chr17	80303530	80303714	185	4	6.25E-06	0.202	0.059	NA
chr12	53183773	53184041	269	4	0.00127	0.195	0.105	NA
chr1	47009527	47010223	697	7	0.00203	0.214	0.021	NA
chr7	1250038	1250756	719	10	0.0002	0.338	0.124	NA
chr7	4175030	4175403	374	4	0.0022	0.086	0.054	NA
chr6	100916916	100917206	291	4	0.00358	-0.293	-0.016	NA
chr1	3229075	3229195	121	2	0.00252	0.227	0.113	NA
chr12	132899503	132900274	772	4	0.0003	-0.237	-0.032	NA
chr6	161575229	161575667	439	7	0.00274	0.178	0.027	NA
chr22	31301703	31302132	430	3	0.00013	0.142	0.058	NA
chr6	32042526	32043029	504	4	0.00267	0.180	0.053	NA
chr8	1398411	1398679	269	2	3.55E-05	0.170	0.085	NA
chr8	1132142	1133105	964	6	0.00041	0.163	0.080	NA
chr5	72593919	72594851	933	5	0.00132	0.252	-0.012	NA
chr20	62031739	62031924	186	2	0.00353	0.190	0.092	NA
chr16	88568209	88568629	421	3	0.00251	0.100	0.029	NA
chr6	32026049	32026891	843	10	3.58E-05	0.210	0.121	NA

chr1	22446397	22446854	458	6	0.00373	0.097	0.020	NA
chr6	32028831	32029216	386	8	0.00301	-0.160	0.068	NA
chr6	32054441	32055146	706	14	0.0002	0.390	0.115	NA
chr12	130501688	130502586	899	11	4.47E-06	0.366	0.212	NA
chr6	31827858	31828373	516	13	0.00108	0.160	0.029	NA
chr6	108479144	108479584	441	6	0.00058	0.207	0.085	NA
chr2	241721320	241722281	962	6	6.96E-06	0.168	0.014	NA
chr6	32050905	32052596	1692	16	0.00013	0.186	0.069	NA
chr10	556470	557109	640	4	0.00045	0.094	0.021	NA
chr1	17004192	17004808	617	5	0.00026	0.125	0.032	NA
chr6	32063991	32065043	1053	34	7.09E-06	0.243	-0.034	NA
chr19	50861774	50862121	348	5	9.57E-05	0.415	0.234	NAPSA
chr2	232348334	232348794	461	4	0.00029	0.431	0.334	NCL
chr19	19626251	19626605	355	6	0.00098	-0.285	-0.137	NDUFA13
chr4	1993350	1993409	60	3	0.00451	0.060	0.042	NELFA
chr17	46125312	46125678	367	7	0.00203	-0.037	-0.009	NFE2L1
chr19	13121571	13125851	4281	15	1.05E-13	0.509	0.324	NFIX
chr2	233877857	233878714	858	6	5.55E-05	0.270	0.047	NGEF
chr20	61884645	61885448	804	8	0.00169	-0.136	-0.006	NKAIN4
chr6	28226885	28227482	598	11	0.00012	-0.360	-0.129	NKAPL
chr10	134600477	134600998	522	12	0.00172	0.017	0.006	NKX6
chr17	33469064	33469488	425	6	0.00332	0.066	0.012	NLE1
chr17	7311030	7311742	713	5	9.12E-05	0.327	0.127	NLGN2
chr2	32490766	32490835	70	2	0.00031	0.328	0.245	NLRC4
chr14	24768737	24769404	668	14	0.00233	-0.020	0.002	NOP9
chr6	32179567	32180641	1075	8	7.25E-06	0.532	0.285	NOTCH4
chr5	32713290	32713464	175	2	0.00353	-0.126	-0.034	NPR3
chr15	72102874	72103908	1035	6	2.92E-06	0.176	0.127	NR2E3
chr15	96866537	96867150	614	3	9.38E-06	0.318	0.213	NR2F2-AS1
chr1	200010708	200011858	1151	9	0.00048	0.214	0.066	NR5A2

chr7	129252859	129253169	311	2	9.38E-05	0.422	0.212	NRF1
chr3	52569026	52569169	144	4	0.00096	-0.133	-0.069	NT5DC2
chr12	96184415	96184580	166	3	0.00348	0.015	0.008	NTN4
chr4	123841683	123841798	116	2	3.03E-07	0.523	0.368	NUDT6
chr19	2268580	2269166	587	3	0.00282	-0.088	-0.023	OAZ1
chr20	45179157	45179413	257	5	1.36E-07	0.477	0.335	OCSTAMP
chr21	34444104	34444382	279	4	0.00289	0.283	0.114	OLIG1
chr11	58036997	58037105	109	2	0.00133	0.265	0.208	OR10W1
chr17	3213470	3213559	90	2	0.00039	0.138	0.081	OR3A4P
chr16	30960055	30960864	810	9	4.55E-06	-0.031	-0.003	ORAI3
chr2	241078542	241078763	222	2	0.00052	0.136	0.102	OTOS
chr17	79801347	79801407	61	2	0.00414	0.116	0.095	P4HB
chr2	147344230	147344418	189	2	0.00108	0.066	0.062	PABPC1P2
chr14	105779532	105779910	379	4	0.00234	0.279	0.158	PACS2
chr14	105851329	105851951	623	4	5.71E-05	0.195	0.082	PACS2
chr7	139760375	139760671	297	2	0.00339	0.211	0.170	PARP12
chr22	44568337	44568812	476	8	3.00E-06	0.199	0.126	PARVG
chr11	31832353	31833477	1125	14	0.0002	0.199	0.035	PAX6
chr6	32155079	32155141	63	3	0.00207	-0.208	-0.090	PBX2
chr4	30724564	30724915	352	3	0.00127	-0.213	-0.188	PCDH7
chr17	79868964	79869463	500	10	7.87E-05	0.016	0.006	PCYT2
chr11	72379867	72380070	204	2	0.00436	0.311	0.259	PDE2A
chr4	656994	657074	81	2	0.00229	-0.208	-0.193	PDE6B
chr1	146649022	146649851	830	5	1.20E-05	0.331	0.240	PDIA3P1
chr7	95225937	95226433	497	7	0.0004	0.189	0.070	PDK4
chr10	6243561	6244010	450	5	0.00072	-0.053	-0.003	PFKFB3
chr14	75423089	75423131	43	3	0.00451	-0.021	-0.016	PGF
chr18	11147146	11148932	1787	9	6.35E-07	-0.314	-0.133	PIEZO2
chr10	104000446	104000831	386	2	0.00242	0.226	0.176	PITX3
chr16	2145598	2145668	71	2	0.00439	0.141	0.111	PKD1

chr11	404596	404762	167	3	0.00235	0.125	0.036	PKP3
chr12	14720249	14721288	1040	12	5.56E-06	-0.142	-0.038	PLBD1
chr3	155421735	155422754	1020	7	0.00014	0.423	0.232	PLCH1
chr17	17108846	17110353	1508	16	2.09E-06	0.185	0.094	PLD6
chr11	112132630	112132648	19	2	0.00419	0.114	0.064	PLET1
chr1	53544373	53544614	242	3	0.00178	0.164	0.077	PODN
chr21	46686908	46687659	752	4	4.50E-06	0.238	0.078	POFUT2
chr8	22102556	22102902	347	7	0.00207	0.013	0.006	POLR3D
chr1	167190178	167190631	454	3	0.00092	0.072	0.030	POU2F1
chr13	79177195	79177520	326	4	0.00363	0.011	0.010	POU4F1
chr17	42018862	42019987	1126	5	6.00E-05	0.152	0.032	PPY
chr17	46799479	46800444	966	9	4.03E-05	-0.256	-0.042	PRAC1
chr10	135160120	135160193	74	2	0.0005	0.318	0.262	PRAP1
chr11	45114570	45114842	273	2	0.00025	0.238	0.178	PRDM11
chr2	44589600	44589694	95	2	0.00239	0.020	0.016	PREPL
chr19	14225172	14225959	788	4	0.00013	0.287	0.094	PRKACA
chr17	27039058	27039523	466	4	0.00246	-0.236	-0.124	PROCA1
chr20	5294595	5295137	543	2	0.00216	0.207	0.159	PROKR2
chr7	29605624	29606349	726	6	7.70E-06	0.275	0.227	PRR15
chr12	11001596	11002609	1014	6	3.78E-11	0.504	0.342	PRR4
chr22	45125218	45126040	823	5	2.90E-09	0.261	0.095	PRR5
chr6	32115964	32116317	354	9	3.68E-05	0.195	0.086	PRRT1
chr16	2866901	2867446	546	4	3.93E-05	0.277	0.173	PRSS21
chr19	696832	696946	115	3	0.00084	0.228	0.009	PRSS57
chr6	31108108	31108541	434	2	0.0014	0.207	0.181	PSORS1C2
chr1	161059267	161059535	269	5	0.00245	0.043	0.030	PVRL4
chr4	122302674	122302680	7	2	0.00441	0.303	0.298	QRFPR
chr22	23487027	23487104	78	3	0.00277	-0.411	-0.216	RAB36
chr20	55925570	55925603	34	3	0.00014	-0.308	-0.278	RAE1
chr7	150039342	150039406	65	3	0.00156	0.173	0.125	RARRES2

chr15	79382708	79383560	853	8	0.00033	0.091	0.014	RASGRF1
chr3	50377836	50379032	1197	13	0.00067	0.159	0.024	RASSF1
chr1	206729553	206729685	133	3	0.00339	0.157	0.096	RASSF5
chr12	86230825	86230957	133	2	0.0018	-0.316	-0.224	RASSF9
chr6	17280740	17281015	276	2	0.00175	0.211	0.196	RBM24
chr2	161265247	161265366	120	4	0.00342	0.238	0.196	RBMS1
chr13	49066868	49066969	102	2	0.00358	0.420	0.357	RCBTB2
chr4	3417677	3418564	888	4	6.65E-05	0.304	0.156	RGS12
chr1	113249654	113249989	336	5	0.00012	0.016	0.009	RHOC
chr1	41131970	41132022	53	2	0.00317	-0.272	-0.211	RIMS3
chr19	39360313	39360961	649	4	0.00108	0.250	0.142	RINL
chr4	190935955	190936289	335	2	0.00019	0.237	0.166	RNA5SP174
chr17	55122747	55123653	907	5	7.93E-05	-0.355	-0.245	RNF126P1
chr10	90342738	90343285	548	9	3.48E-08	0.124	0.068	RNLS
chr12	6586821	6587072	252	4	0.00013	0.072	0.038	RP1-102E24.6
chr17	79430641	79430805	165	2	0.00137	0.087	0.069	RP11-1055B8.7
chr10	135203102	135203200	99	2	0.00064	0.253	0.218	PAOX
chr1	16074114	16074463	350	2	0.00033	0.137	0.108	RP11-169K16.4
chr1	202407864	202408238	375	3	0.00137	0.056	0.034	RP11-175B9.2
chr3	27754049	27755600	1552	11	1.80E-05	0.131	0.067	RP11-222K16.2
chr1	167131969	167132065	97	4	4.66E-05	0.313	0.156	RP11-277B15.1
chr6	58399599	58399793	195	3	0.00074	0.130	0.092	RP11-278J20.2
chr10	134596873	134596989	117	3	0.00225	0.130	0.057	RP11-288G11.3
chr1	146521800	146522878	1079	6	4.69E-07	0.148	0.032	RP11-325P15.1
chr6	41471673	41472657	985	6	0.00066	0.319	0.103	RP11-328M4.3

chr17	59472854	59473124	271	2	0.00047	0.194	0.141	RP11-332H18.4
chr8	136245867	136246255	389	3	4.26E-05	0.567	0.331	RP11-452N4.1
chr16	34441787	34442602	816	4	0.00207	-0.278	-0.140	RP11-488I20.8
chr15	96894342	96895542	1201	6	1.62E-06	0.465	0.405	RP11-522B15.3
chr15	96896940	96897796	857	4	0.00055	0.347	0.185	RP11-522B15.3
chr14	20148103	20148125	23	2	0.00257	0.074	0.017	RP11-597A11.2
chr6	39869689	39869840	152	3	0.00203	0.160	0.107	RP11-61I13.3
chr5	980580	981189	610	6	0.00032	0.498	0.247	RP11-661C8.3
chr1	148853922	148854588	667	4	0.00035	0.062	0.037	RP11-763B22.7
chr11	106698562	106698694	133	3	0.0018	0.131	0.064	RP11-819M15.1
chr12	52215972	52216298	327	5	0.00212	0.098	0.031	RP11-923I11.7
chr9	91267411	91267587	177	2	0.00028	0.457	0.264	RP13-60M5.2
chr6	33868187	33868313	127	3	0.00343	-0.172	0.008	RP3-468B3.2
chr11	30605411	30605797	387	5	0.00242	0.240	0.158	RP5-1024C24.1
chr3	40498475	40499408	934	7	8.65E-08	0.050	0.007	RPL14
chr10	88391658	88391965	308	3	0.00356	-0.235	-0.164	RPL7AP8
chr6	30312580	30313517	938	28	0.00071	0.219	0.008	RPP21
chr1	26869620	26870173	554	2	9.41E-05	0.144	0.134	RPS6KA1
chr19	45999794	46000154	361	5	0.00339	0.088	0.028	RTN2
chr1	25254746	25255003	258	2	0.00144	0.174	0.120	RUNX3
chr1	25256939	25258679	1741	25	2.44E-21	-0.553	-0.276	RUNX3
chr19	39055712	39056216	505	4	0.00327	0.156	0.139	RYR1
chr13	24007965	24008109	145	3	0.00106	0.315	0.084	SACS
chr1	879375	879415	41	3	0.00234	0.154	0.110	SAMD11
chr17	48194500	48195107	608	5	6.88E-05	0.132	0.041	SAMD14

chr6	28557047	28558113	1067	24	2.69E-06	0.452	0.091	SCAND3
chr19	35068221	35069691	1471	7	0.00029	0.284	0.117	SCGB1B2P
chr2	20425981	20426146	166	2	0.00117	0.220	0.115	SDC1
chr19	36485282	36485972	691	7	0.00022	0.286	0.118	SDHAF1
chr17	71434003	71434052	50	3	9.82E-06	0.269	0.167	SDK2
chr16	82044738	82045222	485	8	6.48E-06	0.177	0.101	SDR42E1
chr22	30792674	30793438	765	9	0.00026	-0.017	0.005	SEC14L2
chr10	102731539	102732605	1067	9	0.0003	0.200	0.068	SEMA4G
chr15	74725882	74726520	639	10	0.00272	-0.034	-0.006	SEMA7A
chr10	13391488	13392131	644	4	3.88E-05	0.029	0.020	SEPHS1
chr17	75470810	75471401	592	6	7.10E-05	0.355	0.200	SEPT9
chr17	75316383	75316784	402	3	0.00147	0.404	0.292	SEPT9
chr14	94984140	94984530	391	3	0.00054	0.270	0.207	SERPINA12
chr8	8243576	8243841	266	3	0.00185	-0.036	-0.003	SGK223
chr11	70672365	70673256	892	10	1.71E-08	0.520	0.196	SHANK2
chr7	155599467	155599780	314	3	0.00064	0.312	0.120	SHH
chr15	75747198	75747445	248	2	0.00144	-0.017	-0.004	SIN3A
chr1	2159351	2159373	23	4	0.00284	0.032	0.019	SKI
chr12	129285354	129285402	49	2	0.00369	0.246	0.170	SLC15A4
chr11	798041	799221	1181	7	0.00018	0.106	0.015	SLC25A22
chr1	172717488	172717683	196	2	0.00312	0.104	0.065	SLC25A38P1
chr1	205897159	205897890	732	4	5.67E-05	0.144	0.095	SLC26A9
chr3	125775413	125775693	281	2	0.00241	0.184	0.125	SLC41A3
chr8	142238452	142238504	53	2	0.00474	0.170	0.130	SLC45A4
chr3	11035079	11035357	279	2	0.00037	0.146	0.060	SLC6A1
chr16	87904547	87904580	34	2	0.00054	0.191	0.185	SLC7A5
chr1	41486908	41487437	530	4	3.55E-05	0.251	0.146	SLFNL1
chr5	168271613	168272290	678	8	7.90E-05	-0.356	-0.196	SLIT3
chr16	68482144	68483021	878	10	1.33E-05	0.173	0.034	SMPD3
chr22	31477006	31477330	325	7	0.00224	0.023	0.009	SMTN

chr1	246670199	246670859	661	8	1.45E-05	0.091	0.018	SMYD3
chr20	48599521	48599682	162	5	0.00436	-0.014	0.002	SNAI1
chr7	45144074	45145334	1261	12	1.96E-10	0.238	0.104	SNORA5B
chr3	186505240	186505347	108	2	0.00206	0.323	0.249	SNORA63
chr15	25434289	25435226	938	7	1.08E-06	0.168	0.077	SNORD115
chr15	25337644	25338219	576	3	0.00023	0.135	0.101	SNORD116
chr2	238777587	238777806	220	4	9.43E-07	0.548	0.278	SNORD39.1
chr7	2295998	2296365	368	2	5.80E-05	0.188	0.128	SNX8
chr10	97200911	97201172	262	4	0.00264	0.312	0.171	SORBS1
chr16	1031944	1032266	323	3	0.00294	0.165	0.106	SOX8
chr17	45927392	45930033	2642	13	2.12E-10	-0.376	-0.037	SP6
chr13	24734180	24734318	139	2	0.00085	0.244	0.214	SPATA13
chr3	9233414	9234107	694	3	4.71E-07	0.251	0.164	SRGAP3-AS2
chr1	54724018	54724125	108	2	0.00061	0.080	0.056	SSBP3
chr12	109205201	109205487	287	3	7.26E-05	0.154	0.043	SSH1
chr3	186647950	186648285	336	4	5.80E-05	0.260	0.060	ST6GAL1
chr7	116659609	116660240	632	4	0.00014	0.358	0.101	ST7
chr17	37380697	37381273	577	3	1.08E-08	-0.440	-0.166	STAC2
chr4	184922382	184922406	25	2	0.00457	-0.369	-0.361	STOX2
chr17	56429567	56430170	604	8	0.0015	-0.030	0.002	SUPT4H1
chr20	58507171	58507232	62	3	1.99E-05	-0.314	-0.195	SYCP2
chr6	152958649	152959047	399	4	0.00102	0.275	0.072	SYNE1
chr17	76164111	76164396	286	4	0.00175	0.294	0.157	SYNGR2
chr12	118810744	118811074	331	10	0.00096	0.021	0.005	TAOK3
chr6	32821039	32821814	776	18	0.00013	-0.037	-0.008	TAP1
chr4	37978758	37979303	546	3	0.001	0.036	0.022	TBC1D1
chr17	77923971	77924733	763	8	0.0003	0.242	0.118	TBC1D16
chr13	76055871	76056030	160	3	0.00172	0.026	0.012	TBC1D4
chr17	80882894	80883274	381	3	0.00257	0.095	0.064	TBCD
chr17	80898443	80898986	544	5	0.00151	0.389	0.100	TBCD

chr16	2021690	2022307	618	10	6.71E-05	-0.031	-0.007	TBL3
chr20	62694736	62695569	834	4	0.00036	-0.251	-0.055	TCEA2
chr20	62687969	62687986	18	2	0.0029	0.301	0.259	TCEA2
chr6	31129329	31130398	1070	18	6.27E-05	0.186	0.067	TCF19
chr11	67809980	67810742	763	7	0.00031	0.142	0.058	TCIRG1
chr2	105946563	105946882	320	4	0.00213	0.222	0.034	TGFBRAP1
chr20	35201208	35202059	852	7	0.00037	0.182	0.036	TGIF2
chr17	38219361	38220298	938	5	0.0003	-0.316	-0.062	THRA
chr17	1613017	1613239	223	2	0.0003	0.223	0.118	TLCD2
chr10	98273510	98274264	755	8	0.00062	0.081	0.021	TLL2
chr1	223316219	223316927	709	9	0.00017	0.114	0.025	TLR5
chr3	129407365	129407548	184	5	7.60E-06	0.198	0.170	TMCC1
chr17	53800372	53800554	183	3	0.00475	0.334	0.246	TMEM100
chr1	1476269	1476845	577	4	0.00017	-0.152	0.023	TMEM240
chr2	120189622	120190297	676	5	8.10E-07	-0.209	0.014	TMEM37
chr1	1850414	1851416	1003	15	0.00039	0.121	0.021	TMEM52
chr16	426713	427084	372	3	0.00117	0.073	0.030	TMEM8A
chr6	31544931	31545898	968	10	0.00018	0.163	0.089	TNF
chr11	128812462	128813470	1009	15	1.27E-07	0.378	0.099	TP53AIP1
chr1	3662685	3663705	1021	7	0.00011	-0.154	-0.034	TP73-AS1
chr3	127311038	127311169	132	3	0.00024	0.334	0.100	TPRA1
chr3	127299170	127299831	662	4	0.00033	0.212	0.076	TPRA1
chr13	45945197	45945362	166	3	0.00089	0.168	0.140	TPT1-AS1
chr17	7836533	7836610	78	3	0.00304	0.181	0.064	TRAPPC1
chr6	116866658	116867006	349	3	0.00406	0.293	0.233	TRAPPC3L
chr8	141248988	141249094	107	2	0.00058	0.180	0.147	TRAPPC9
chr19	59055873	59056208	336	2	0.00399	0.008	0.005	TRIM28
chr1	33646902	33647028	127	2	0.00371	-0.015	-0.002	TRIM62
chr20	33681070	33681223	154	2	0.00225	-0.173	-0.158	TRPC4AP
chr17	3438857	3439372	516	7	0.00055	0.238	0.063	TRPV3

chr8	143436137	143436205	69	2	0.00091	0.181	0.142	TSNARE1
chr1	231663393	231663886	494	2	0.00135	0.290	0.154	TSNAX
chr11	2321770	2322050	281	8	0.0006	0.240	0.087	TSPAN32
chr11	2422520	2422730	211	10	0.00372	-0.051	-0.003	TSSC4
chr2	179396688	179397513	826	7	1.49E-05	0.095	0.047	TTN-AS1
chr15	41851964	41851969	6	2	0.00452	0.034	0.031	TYRO3
chr16	4665248	4666047	800	5	2.06E-07	-0.266	-0.136	UBALD1
chr17	16282382	16282434	53	3	0.0008	0.127	0.090	UBB
chr5	176292536	176292607	72	3	0.00093	0.277	0.184	UNC5A
chr11	67764320	67765720	1401	7	1.41E-06	0.276	0.096	UNC93B1
chr15	63893412	63893530	119	2	0.0015	0.030	0.017	USP3-AS1
chr1	160388964	160389104	141	2	0.00464	0.127	0.102	VANGL2
chr10	135050326	135051022	697	17	0.00087	0.096	0.009	VENTX
chr3	11763045	11763340	296	3	0.00125	0.278	0.225	VGLL4
chr8	145654780	145654854	75	2	0.00408	-0.212	-0.182	VPS28
chr6	33235504	33235977	474	7	0.00055	0.163	0.059	VPS52
chr16	699218	699775	558	7	2.74E-05	-0.026	0.000	WDR90
chr16	703597	704060	464	5	0.00038	-0.122	-0.004	WDR90
chr16	678562	678891	330	2	0.00016	0.201	0.116	WFIKKN1
chr22	46366859	46368025	1167	5	3.00E-10	0.662	0.446	WNT7B
chr16	69958626	69958964	339	4	0.00242	0.372	0.274	WWP2
chr19	7694013	7694688	676	11	0.00023	-0.035	-0.005	XAB2
chr6	169689773	169689939	167	3	2.77E-08	0.482	0.184	XXyac-YX65C7_A.3
chr6	31370412	31371967	1556	43	0.00011	0.094	0.002	Y_RNA.22
chr1	207224102	207225647	1546	12	1.12E-08	0.200	0.077	YOD1
chr6	31869301	31869443	143	3	0.00448	0.019	0.015	ZBTB12
chr6	33286540	33287248	709	8	0.00028	0.225	0.087	ZBTB22
chr14	105268492	105269174	683	5	0.0006	-0.373	-0.226	ZBTB42
chr7	1199841	1200356	516	8	0.00214	0.024	0.009	ZFAND2A

chr16	89788035	89788316	282	2	0.00046	0.013	0.009	ZNF276
chr1	200378667	200379235	569	6	0.00215	-0.103	-0.015	ZNF281
chr19	58596723	58597097	375	3	0.00254	0.157	0.095	ZSCAN18

S3 Table: Summary of the 28 DNA regions differentially methylated between donors with and without asthma when pronase and bronchial brush isolated cells are combined in a single analysis.

Chromosome Location	Start Location	End Location	Region Width	Number of CpGs	Minimum FDR	Max. difference in β value	Mean difference in β value	Overlapping Promoters
chr7	1581583	1582222	640	4	5.88E-07	-0.15	-0.03	AC093734.1
chr5	2754240	2755158	919	6	0.00202364	0.24	0.14	C5orf38-001
chr11	64951536	64952354	819	6	0.000402	0.15	0.09	CAPN1
chr13	36871646	36872346	701	13	0.000402	-0.05	-0.03	CCDC169, CCDC169-SOHLH2, SOHLH2, SOHLH2
chr2	130897120	130897302	183	3	0.001548832	0.21	0.16	CCDC74B
chr6	*291687*	*292823*	*1137*	*9*	*3.97E-05*	*0.17*	*0.13*	*DUSP22*
**chr10**	**123353298**	**123354171**	**874**	**13**	**4.45E-10**	**0.15**	**0.12**	**FGFR2**
**chr6**	**101846409**	**101847706**	**1298**	**18**	**1.30E-10**	**0.28**	**0.17**	**GRIK2**
**chr12**	**6881118**	**6882083**	**966**	**9**	**0.000360835**	**-0.18**	**-0.10**	**LAG3**
**chr17**	**66194621**	**66194797**	**177**	**5**	**0.002748163**	**0.16**	**0.12**	**LOC440461**
**chr13**	**36050158**	**36051073**	**916**	**9**	**0.000402**	**0.21**	**0.18**	**MAB21L1, NBEA-203**

chr15	23810861	23811205	345	2	0.002630989	0.10	0.09	MKRN3
chr10	530635	531584	950	12	0.000289991	-0.17	-0.09	NA
chr15	70326224	70326230	7	2	0.003989859	0.22	0.18	NA
chr17	45944339	45944464	126	3	0.000402	0.11	0.06	NA
chr6	30122523	30122593	71	4	0.001604623	0.23	0.19	NA
chr6	169567826	169569137	1312	6	0.000909561	-0.10	-0.03	NA
**chr7**	**113722758**	**113723162**	**405**	**4**	**0.001236086**	**0.24**	**0.21**	**NA**
chr7	4832112	4832535	424	5	0.000293169	0.16	0.13	NA
**chr8**	**1906338**	**1906498**	**161**	**3**	**0.000486283**	**0.28**	**0.22**	**NA**
chr4	72897565	72898212	648	6	0.000326774	-0.14	-0.11	NPFFR2
**chr17**	**45927392**	**45928077**	**686**	**3**	**0.000563631**	**0.16**	**0.13**	**SP6**
chr6	134210138	134210279	142	5	0.004776081	-0.20	-0.09	TCF21, RP3-323P13.2
chr11	59633874	59634276	403	4	0.000866335	0.27	0.17	TCN1
chr15	*91473059*	*91473569*	*511*	*8*	*1.19E-08*	*-0.17*	*-0.07*	*UNC45A*
chr5	135415258	135416613	1356	15	2.35E-06	0.20	0.14	VTRNA2
chr3	113160183	113160637	455	8	0.00032984	0.12	0.09	WDR52
chr17	48912483	48912764	282	8	0.003513907	0.16	0.09	WFIKKN2
chr20	5485144	5485873	730	9	2.37E-76	0.28	0.18	LINC00654
chr17	4804674	4804838	165	2	2.97E-21	-0.17	-0.16	CHRNE, C17orf107
chr6	101846409	101847706	1298	18	9.24E-33	-0.33	-0.21	GRIK2
chr22	17680477	17680706	230	8	7.21E-19	0.18	0.11	CECR1
chr2	15804917	15805012	96	3	2.28E-19	-0.13	-0.08	NA
chr6	134210279	134210307	29	2	1.15E-18	0.09	0.08	TCF21

chr6	29520965	29521803	839	31	1.77E-30	0.23	0.13	OR2I1P
chr6	33287809	33289280	1472	32	2.85E-55	0.31	0.13	DAXX
chr1	40105664	40105764	101	5	1.93E-19	-0.16	-0.07	HEYL

S4 Table: Comparison of regions overlapping between the combined analysis and the analyses on distinct isolation techniques.

Region Gene	Combined Region:			
	Size	Max beta difference	Mean beta difference	Direction of change
**FGFR2**	**Equal**	**Smaller**	**Smaller**	**Same**
**GRIK2**	**Larger**	**Smaller**	**Smaller**	**Same**
**LAG3**	**Equal**	**Smaller**	**Smaller**	**Same**
**LOC440461**	**Smaller**	**Smaller**	**Larger**	**Same**
**MAB21L1, NBEA-203**	**Smaller**	**Smaller**	**Smaller**	**Same**
**NA**	**Smaller**	**Smaller**	**Smaller**	**Same**
**NA**	**Equal**	**Smaller**	**Smaller**	**Same**
**SP6**	**Smaller**	**Smaller**	**Larger**	**Opposite**
UNC45A	*Equal*	*Larger*	*Larger*	*Same*
DUSP22	*Smaller*	*Smaller*	*Smaller*	*Same*
